# Study on the association between sleep quality and anxiety–depression symptoms in school-aged children

**DOI:** 10.3389/fpubh.2026.1927475

**Published:** 2026-10-20

**Authors:** Jiao Chen, Yiling Liu, Qin Liu, Fan Zhang

**Affiliations:** Research Center for Medicine and Social Development, College of Public Health, Chongqing Medical University, Chongqing, China

**Keywords:** anxiety, depression, longitudinal study, school-aged children, sleep quality

## Abstract

**Background:**

To analyze the short-term longitudinal associations between sleep-related problems and anxiety, depression and the coexistence of anxiety and depression in school-aged children, and to provide the epidemiological evidence for mental health surveillance and risk assessment.

**Methods:**

This study was part of a child and adolescent growth and development cohort in an urban district of Chongqing. Participants were recruited from grade 1–3 at three local primary schools. A total of 1,529 children (782 boys and 747 girls; average age = 8.00 ± 1.0 years) were included who completed two surveys. Sleep, anxiety, and depressive symptoms were assessed using the PSQI-5, GAD-7, and CDI-S, respectively. Binary logistic regression and multiple linear regression were used to examine the associations between baseline sleep levels, sleep change values (ΔPSQI), and follow-up emotional outcomes. Missing covariates were handled via multiple imputation.

**Results:**

The total scores of PSQI-5, GAD-7, and CDI-S at the follow-up were all lower than those at the baseline (*p* < 0.001). After adjusting for confounding factors and baseline emotional levels, a higher baseline PSQI independently predicted subsequent anxiety (OR = 1.144, *p* < 0.001), depression (OR = 1.097, *p* = 0.013), and coexisting symptoms (OR = 1.146, *p* = 0.003). The increases in ΔPSQI was positively associated with the aggravation of emotional symptoms (all *p* < 0.001).

**Conclusion:**

Long-term poor sleep and decreased sleep quality may increase the risk of internalizing problems in school-aged children.

## 1 Introduction

The school-aged period typically refers to the stage from the time when children enter primary school until the onset of puberty. According to the age classification in pediatric medicine and the common definitions used in child health care research, school-age children generally range from 6 to 12 years old and are in the primary school stage (1, 2). This period is crucial for the development of emotional, behavioral, and social functions, and also represents a critical window during which internalizing problems such as anxiety and depression gradually emerge and begin to affect learning and daily life. According to the Global Burden of Disease (GBD) study, an estimated 9.33 million and 38.20 million cases of depression and anxiety, respectively, occurred globally among children aged 5–14 years in 2021 (3). National clinical data from China indicate that the prevalence of anxiety and depressive disorders among children and adolescents aged 6–16 years is 4.7 and 3.0%, respectively (4). These figures underscore that emotional health problems represent a substantial population-level burden in school-aged children and adolescents.

From an epidemiological perspective, depression and anxiety are not rare in children and adolescents, and their prevalence varies across age groups and populations. According to the latest data from the National Survey of Children’s Health (NSCH) in the United States, the prevalence of anxiety and depressive disorders among children aged 3–17 years is 7.1 and 3.2%, respectively (5). Analyses of global burden of disease data have shown that the burden of depressive disorders among individuals aged 10–24 years has generally increased from 1990 to 2019 (6). Meanwhile, evidence supporting cognitive-behavioral therapy for childhood anxiety and psychosocial interventions for adolescent depression continues to accumulate (7, 8). Therefore, identifying characteristics that are readily observable and longitudinally trackable in school settings before symptoms progress to full-blown clinical disorders is of practical importance.

Anxiety and depression during childhood and adolescence do not always occur independently but frequently present as co-occurring conditions. Existing studies have shown that there is a significant overlap in symptom manifestations, development processes, and risk factors between the two, and the presence of one symptom dimension often increases the possibility of the continuation or development of another symptom dimension (9, 10). Therefore, paying attention to both single symptoms and coexisting states at the school stage is more in line with the manifestation of children’s emotional problems in reality.

Sleep is a fundamental process linking physiological regulation and mental health. More and more studies suggest that there is not a simple one-way relationship between sleep and internalizing problems, but rather a more likely continuous and intertwined interaction process (11). In Chinese adolescents, declines in sleep quality have been associated with subsequent increases in anxiety and depressive symptoms (12). Existing research has underscored that there may also be a bidirectional relationship between sleep and anxiety/depression. Children and adolescents with symptoms of anxiety and depression often experience insufficient sleep, and insufficient sleep may further trigger or exacerbate anxiety and depression (13–15). These findings indicate the necessity of continuous attention to sleep in the study of emotional problems among school populations.

In addition to individual physiological factors, sleep is embedded in a wider family and school environment. In the daily life environment jointly formed by school and family, sleep is influenced by multiple lifestyle and environmental factors. Studies have indicated that the sense of belonging and acceptance among children and adolescents in school, as well as their relationships with teachers and classmates, are related to their sleep health status (16). This suggests that sleep reflects not only an individual’s rest status but also the combined effects of family environment, school life, and daily behavioral patterns.

Although previous research has extensively examined the relationship between sleep and anxiety/depression in children and adolescents, several gaps remain. School-aged children are in a developmental stage characterized by ongoing maturation of sleep habits, emotion regulation, and school adaptation. Their sleep-related problems and emotional symptom profiles may differ from those of adolescents. Although the bidirectional association between sleep and emotional symptoms has been widely recognized, examining the unidirectional predictive effect of sleep problems on subsequent emotional symptoms also has independent scientific value. If sleep problems can independently predict the emergence of children’s subsequent emotional symptoms, they can serve as easily identifiable and observable indicators at home and school, and provide quantitative reference for school mental health observations. Therefore, this study, based on the growth and development cohort of children and adolescents in a certain urban area of Chongqing, focuses on analyzing the unidirectional longitudinal association between sleep-related problems and emotional symptoms in school-aged children. The aim is to explore the prospective relationship between the two from the perspective of epidemiological risk identification, rather than establishing a predictive tool with individual screening or diagnostic functions, nor involving the inference of dynamic causal relationships or bidirectional interaction relationships.

Based on the existing understanding of the relationship between sleep and emotion regulation and the prevalence of anxiety and depression comorbidity in children and adolescents, this study aims to verify the following hypotheses:

*H1: The higher the severity of sleep-related problems in school-age children, the greater the possibility of anxiety symptoms, positive depressive symptoms, and their coexistence.*

*H2: The associations between specific sleep dimensions and subsequent emotional outcomes may differ.*

## 2 Materials and methods

### 2.1 Research subjects

This study is based on a growth and development cohort of children and adolescents in a certain urban area of Chongqing. In this urban area, three primary schools that met the survey requirements and agreed to participate were selected, covering students from grades one to three. Follow-up surveys were conducted every 6 months. The first assessment of sleep, anxiety, depressive symptoms, and covariates conducted from May to June 2024 served as the baseline survey for this study; the survey conducted from November to December 2024 served as the follow-up survey. No intervention was implemented between these two time points.

A total of 1,620 valid questionnaires were collected at the baseline, and 1,744 valid questionnaires were collected during the follow-up. The follow-up sample size was higher than that at baseline because some students were absent or had incomplete scale information during the baseline survey and were not included in the valid sample at the baseline, but completed valid questionnaires during the follow-up survey. A total of 1,529 children who completed both the baseline and follow-up surveys and underwent assessment of sleep, anxiety, and depression symptom scales were selected as the longitudinal analysis sample. The inclusion and follow-up procedures of the research subjects are detailed in Figure 1.

**Figure 1:**
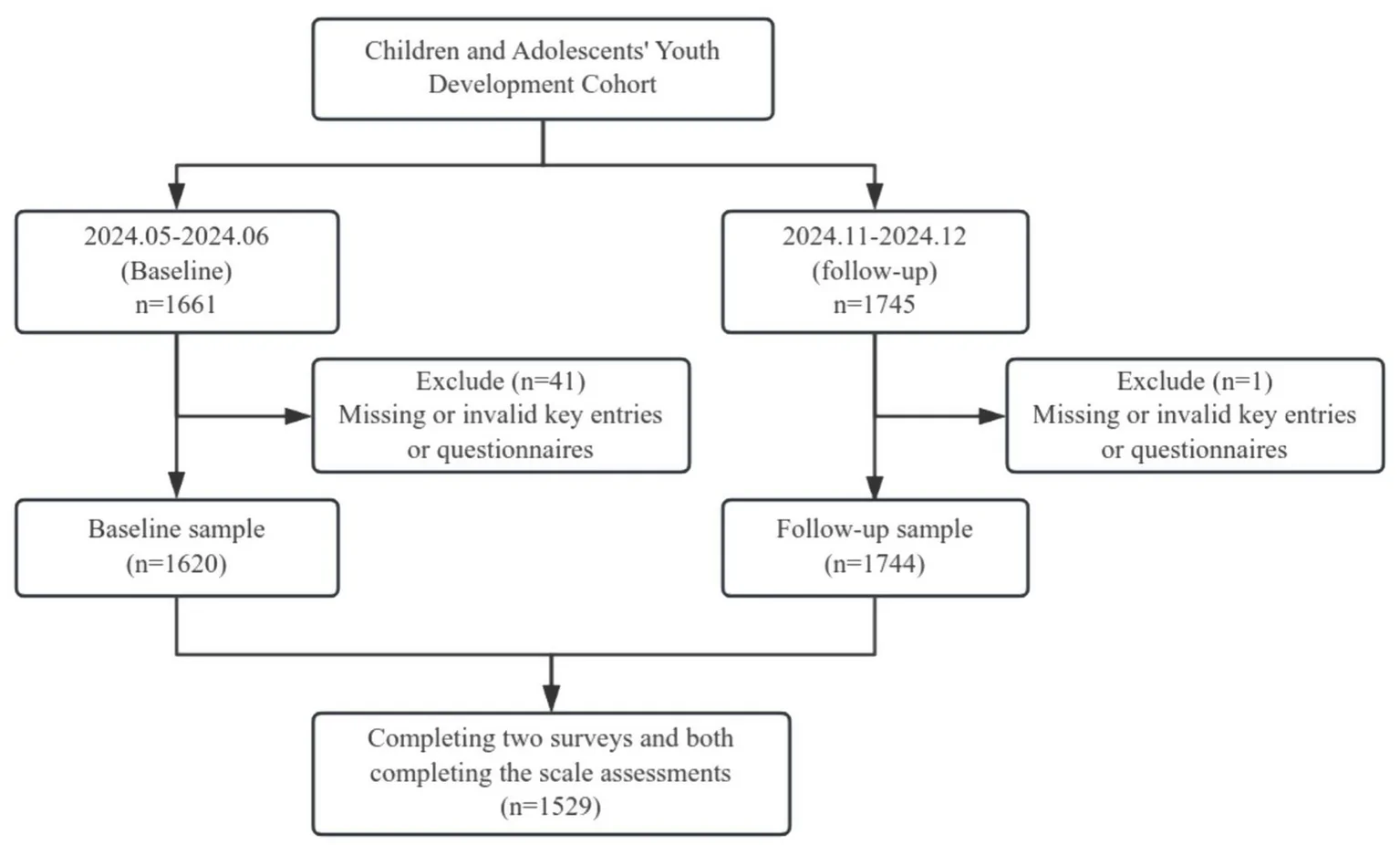
Participation flow chart.

All research subjects collected data using an anonymous numbering method. The inclusion criteria for the research subjects were: (i) completed the assessment of sleep, anxiety, and depression symptom scales in the current survey; (ii) voluntarily participated and signed the informed consent form. Exclusion criteria were: (i) missing key items in the sleep, anxiety, or depression scales, resulting in inability to score or inability to determine the outcome; (ii) questionnaires with obvious logical errors, judged as invalid questionnaires by the researchers.

### 2.2 Measures

Data were collected using structured questionnaires. The child self-report questionnaires were administered to assess sleep, anxiety, and depressive symptoms. The maternal questionnaires were used to collect information on child and family characteristics, as detailed below.

#### 2.2.1 Basic information

The basic information of the research subjects was collected via a maternal questionnaire. The content included the social demographic characteristics of the children, the social economic characteristics of families, family structure, health conditions, and some behavioral habits, specifically covering the grade, sex, residence, family structure, father’s education level, mother’s education level, monthly household income per capita, whether the child had chronic diseases, the frequency of eating after 9 p.m., and whether the child goes to bed with the light on, etc.

#### 2.2.2 Sleep-related issues

The Pittsburgh Sleep Quality Index (PSQI) relevant items were used to assess the sleep status over the past month. The PSQI was developed by Buysse et al. (17) and is a widely used subjective sleep assessment tool internationally. The complete PSQI includes items on bedtime, wake-up time, actual sleep duration, and sleep efficiency, and requires high accuracy in time recall and self-report. Through initial communication with the students, we found that for the research subjects in the primary school stage, the questionnaire should be completed in a collective classroom environment and completed within a limited time; otherwise, there may be significant errors in the recall of specific sleep time and sleep efficiency. In addition to the problems discovered during on-site communication, the research team also conducted literature review and group discussion. Previous studies have shown that the PSQI can be simplified or optimized according to the research purpose, but the construction basis, applicable subjects, and scoring explanations of different simplified forms are not completely consistent (18, 19). Therefore, our team retained the five dimensions that are most directly related to the research question and most reliably reported by children, thereby constructing the PSQI-5 comprehensive index. This index includes subjective sleep quality, sleep onset time, sleep disorders, use of hypnotic drugs, and daytime functional impairment. Each dimension is scored from 0 to 3 according to the scoring rules of the original scale, with a total score ranging from 0 to 15, and higher scores indicate more severe sleep-related problems. Its results are not equivalent to the total score of the complete PSQI, nor do they directly correspond to the complete PSQI thresholds under traditional clinical standards. In this study, the Cronbach’s alpha coefficient of PSQI-5 at the baseline and follow-up surveys was 0.495 and 0.540 respectively, indicating relatively low internal consistency and possible measurement errors. However, the PSQI-5 is composed of different sleep dimensions, and each dimension reflects different sleep characteristics, and the internal consistency coefficient cannot fully evaluate the full use of this comprehensive indicator.

#### 2.2.3 Anxiety symptoms

The Generalized Anxiety Disorder-7 (GAD-7) was used to assess the anxiety symptom level of the research subjects in the past 2 weeks. GAD-7 consists of 7 items, each scored 0–3 based on the frequency of symptom occurrence, with a total score range of 0–21, and higher scores indicate greater anxiety severity (20). Previous studies have shown that GAD-7 has good structural validity and measurement stability in Chinese adolescents and other young people, and can be used for screening and stratification of anxiety symptoms at the population level (21–23). In this study, a GAD-7 total score ≥5 was defined as screening-positive for anxiety.

#### 2.2.4 Depression symptoms

The depression symptoms of the research subjects in the past 2 weeks were evaluated using the Short Form of the Children’s Depression Inventory (CDI-S). The CDI-S is a 10-item short form of the original Children’s Depression Inventory, with each item consisting of three statements increasing in symptom severity, scored 0–2, yielding a total score from 0 to 20, and higher scores indicate more severe depressive symptoms (24, 25). Previous studies have shown that CDI-S has certain structural validity and discriminant validity, and has been used in studies of Chinese children and adolescents (26, 27). In combination with the school population survey scenario of this study, the same standard was adopted, that is, a CDI-S total score ≥7 was defined as screening-positive for depression (28).

#### 2.2.5 Co-occurrence of anxiety and depression symptoms

Based on the screening threshold of the scale, this study defined those who met both GAD-7 total score ≥5 and CDI-S total score ≥7 in the same survey as having co-occurring anxiety and depression symptoms.

All the definitions of the above scales reflect the status in the context of screening and are not intended for clinical diagnosis.

### 2.3 Statistical analysis

The data were double-entered using EpiData and analyzed with IBM SPSS Statistics 26.0. Missing covariate data were handled using multiple imputation (MI) with fully conditional specification (FCS), generating 20 imputed datasets. To assess the robustness of the imputation results, we performed a sensitivity analysis restricted to the 1,405 children with complete covariate data, comparing whether the association directions and magnitudes of the main exposure variables and subsequent emotional outcomes were consistent with the results of the main analysis. All tests were two-sided, with *p* < 0.05 considered statistically significant.

Continuous variables were described using the median and interquartile range (IQR), while categorical variables were expressed as frequencies (*n*) and percentages (%). Group comparisons were made using the Mann–Whitney *U* or Kruskal–Wallis *H* tests for continuous variables, and the chi-square or Fisher’s exact test for categorical variables. Binary logistic regression and PSQI-5 quartile-based analyses were used to examine the association between baseline sleep and follow-up emotional outcomes.

The sleep change index, ΔPSQI, was calculated as the follow-up PSQI-5 minus the baseline PSQI-5. In the model with follow-up anxiety and depression coexistence as the outcome, ΔPSQI and baseline PSQI-5 were entered simultaneously to estimate their independent associations with the outcome. For brevity, the full results of all covariates are presented in Tables 1–5 of the main text. Other tables (Tables 6–12) only include the main exposure variables and key covariates, and the full results of all covariates can be found in the Supplementary Tables S1–S15.

**Table 1:** Participant characteristics and distribution of sleep-related problems and emotional symptom indicators at baseline and follow-up.

Variable	Baseline (*n* = 1,620)		Follow-up (*n* = 1,744)	
	Median (IQR)	*n* (%)	Median (IQR)	*n* (%)
Sleep-related problems				
PSQI-5 total scores	4.00 (3.00)		4.00 (3.00)	
Subjective sleep quality	1.00 (1.00)		1.00 (1.00)	
Sleep latency	1.00 (2.00)		1.00 (1.00)	
Sleep disturbance	1.00 (0.00)		1.00 (0.00)	
Use of sleep medication	0.00 (0.00)		0.00 (0.00)	
Daytime dysfunction	2.00 (1.00)		2.00 (1.00)	
Depressive symptoms				
CDI-S total scores	4.00 (4.00)		4.00 (4.00)	
<7		1,259 (77.7)		1,411 (80.9)
≥7		361 (22.3)		333 (19.1)
Anxiety symptoms				
GAD-7 total scores	3.00 (5.00)		3.00 (5.00)	
<5		990 (61.1)		1,120 (64.2)
≥5		630 (38.9)		624 (35.8)
Co-occurring anxiety and depression symptoms				
No		1,352 (83.5)		1,515 (86.9)
Yes		268 (16.5)		229 (13.1)
Sociodemographic characteristics				
Sex				
Male		839 (51.8)		895 (51.3)
Female		781 (48.2)		849 (48.7)
Age (years)	8.00 (2.00)		8.00 (1.00)	
Weight (kg)	26.40 (7.50)		28.80 (9.10)	
Grade				
Grade 1		435 (26.9)		-
Grade 2		523 (32.3)		534 (30.6)
Grade 3		662 (40.9)		577 (33.1)
Grade 4		-		633 (36.3)
School				
School A		353 (21.8)		530 (30.4)
School B		878 (54.2)		858 (49.2)
School C		389 (24.0)		356 (20.4)
Residence				
Urban		1,413 (95.5)		1,538 (95.8)
Rural		67 (4.5)		68 (4.2)
Father’s education level				
Primary school or below		21 (1.4)		20 (1.2)
Junior high school		227 (15.3)		237 (14.8)
High school or technical secondary school		380 (25.7)		400 (24.9)
Bachelor or associate degree		772 (52.2)		861 (53.6)
Master’s degree or above		80 (5.4)		88 (5.5)
Mother’s education level				
Primary school or below		14 (0.9)		15 (0.9)
Junior high school		223 (15.1)		230 (14.3)
High school or technical secondary school		390 (26.4)		405 (25.2)
Bachelor or associate degree		794 (53.6)		886 (55.2)
Master’s degree or above		59 (4.0)		70 (4.4)
Monthly household income per capita (yuan)				
<2000		47 (3.2)		48 (3.0)
2000–4,000		300 (20.3)		311 (19.4)
4,000–6,000		247 (16.7)		254 (15.8)
6,000–8,000		236 (15.9)		263 (16.4)
8,000–10,000		250 (16.9)		281 (17.5)
>10,000		400 (27.0)		449 (28.0)
Family structure				
Two-parent family		1,280 (86.5)		1,386 (86.3)
Single mother family		131 (8.9)		142 (8.8)
Single father family		37 (2.5)		46 (2.9)
Both parents absent		32 (2.2)		32 (2.0)
Only child				
Yes		570 (38.6)		631 (39.4)
No		908 (61.4)		972 (60.6)
Father/mother deceased				
Yes		11 (0.7)		10 (0.6)
No		1,469 (99.3)		1,596 (99.4)
Parental divorce				
Yes		425 (28.7)		499 (31.1)
No		1,055 (71.3)		1,107 (68.9)
Chronic disease				
No		1,584 (97.8)		1,705 (97.8)
Yes		36 (2.2)		39 (2.2)
Eating after 9 p.m.				
Never		272 (18.4)		297 (18.5)
Occasionally		1,165 (78.7)		1,259 (78.4)
Every day		43 (2.9)		50 (3.1)
Prefer sleeping with light on				
Yes		193 (13.0)		207 (12.9)
No		1,287 (87.0)		1,399 (87.1)

The composition ratios were calculated based on the number of valid samples for each variable. The comparison between the baseline and follow-up scales was conducted using the Wilcoxon signed-rank test: PSQI-5 (*Z* = −5.682, *p* < 0.001), GAD-7 (*Z* = −4.038, *p* < 0.001), and CDI-S (*Z* = −4.578, *p* < 0.001).

**Table 2:** Longitudinal association between baseline sleep-related problems and follow-up positive anxiety symptoms (*n* = 1,529).

Variable	Classification	Model 1		Model 2		Model 3		Model 4	
		OR (95% CI)	*p*	OR (95% CI)	*p*	OR (95% CI)	*p*	OR (95% CI)	*p*
Baseline PSQI-5		1.382 (1.308–1.462)	**<0.001**	1.367 (1.292–1.447)	**<0.001**	1.368 (1.292–1.447)	**<0.001**	1.144 (1.071–1.222)	**<0.001**
Sex (Male = 0)	Female			0.737 (0.587–0.924)	**0.008**	0.728 (0.577–0.918)	**0.007**	0.709 (0.555–0.906)	**0.006**
School (School A = 0)	School B			0.964 (0.710–1.307)	0.811	0.961 (0.708–1.305)	0.801	0.922 (0.667–1.276)	0.625
	School C			0.823 (0.548–1.238)	0.350	0.827 (0.549–1.247)	0.364	0.824 (0.533–1.272)	0.382
Grade (Grade 1 = 0)	Grade 2			0.823 (0.609–1.114)	0.208	0.826 (0.607–1.124)	0.224	0.946 (0.685–1.307)	0.737
	Grade 3			0.797 (0.593–1.071)	0.132	0.825 (0.596–1.142)	0.247	0.923 (0.654–1.303)	0.650
Residence (urban = 0)	Rural			1.126 (0.618–2.051)	0.697	1.133 (0.620–2.071)	0.685	1.201 (0.643–2.241)	0.565
Parental divorce (no = 0)	Yes			1.124 (0.825–1.530)	0.460	1.139 (0.835–1.552)	0.412	1.138 (0.819–1.583)	0.441
Father/mother deceased (no = 0)	Yes			0.991 (0.325–3.023)	0.987	0.927 (0.305–2.822)	0.893	1.174 (0.373–3.690)	0.784
Family structure (two-parent family = 0)	Single mother family			1.312 (0.866–1.987)	0.200	1.318 (0.868–2.002)	0.196	1.119 (0.716–1.750)	0.622
	Single father family			1.456 (0.712–2.974)	0.303	1.408 (0.695–2.855)	0.342	1.272 (0.603–2.681)	0.527
	Both parents absent			1.390 (0.620–3.114)	0.424	1.359 (0.603–3.062)	0.460	1.275 (0.529–3.070)	0.588
Only child (no = 0)	Yes			0.891 (0.696–1.141)	0.361	0.883 (0.689–1.132)	0.328	0.844 (0.649–1.097)	0.204
Father’s education level (primary school or below = 0)	Junior high school			0.777 (0.327–1.847)	0.568	0.799 (0.329–1.937)	0.618	0.866 (0.345–2.177)	0.760
	High school or technical secondary school			0.596 (0.255–1.393)	0.232	0.616 (0.257–1.473)	0.275	0.668 (0.267–1.667)	0.386
	Bachelor or associate degree			0.503 (0.213–1.187)	0.117	0.518 (0.215–1.250)	0.143	0.541 (0.214–1.366)	0.193
	Master’s degree or above			0.599 (0.214–1.675)	0.328	0.597 (0.211–1.688)	0.330	0.672 (0.223–2.023)	0.479
Mother’s education level (primary school or below = 0)	Junior high school			0.845 (0.251–2.850)	0.786	0.866 (0.255–2.942)	0.818	0.574 (0.162–2.039)	0.390
	High school or technical secondary school			1.173 (0.350–3.924)	0.796	1.190 (0.353–4.011)	0.778	0.859 (0.242–3.055)	0.814
	Bachelor or associate degree			1.050 (0.309–3.563)	0.938	1.077 (0.315–3.685)	0.905	0.744 (0.207–2.676)	0.650
	Master’s degree or above			1.176 (0.306–4.519)	0.813	1.199 (0.311–4.617)	0.792	0.840 (0.207–3.405)	0.806
Monthly household income per capita (yuan) (<2000 = 0)	2000–4,000			1.120 (0.563–2.229)	0.747	1.160 (0.576–2.338)	0.677	1.178 (0.569–2.437)	0.659
	4,000–6,000			1.061 (0.517–2.177)	0.871	1.093 (0.526–2.273)	0.811	1.062 (0.496–2.274)	0.876
	6,000–8,000			1.216 (0.596–2.483)	0.591	1.258 (0.610–2.597)	0.534	1.355 (0.637–2.882)	0.430
	8,000–10,000			0.921 (0.447–1.897)	0.823	0.948 (0.455–1.974)	0.886	1.024 (0.478–2.195)	0.951
	>10,000			1.083 (0.532–2.207)	0.825	1.114 (0.541–2.293)	0.770	1.140 (0.537–2.421)	0.732
Chronic disease (no = 0)	Yes					1.036 (0.483–2.221)	0.927	1.055 (0.471–2.363)	0.896
Weight (kg)						0.995 (0.976–1.014)	0.588	0.990 (0.970–1.010)	0.332
Prefer sleeping with light on (no = 0)	Yes					1.029 (0.731–1.448)	0.872	0.965 (0.671–1.388)	0.847
Eating after 9 p.m. (never = 0)	Occasionally					0.818 (0.603–1.109)	0.195	0.831 (0.602–1.146)	0.259
	Every day					1.149 (0.537–2.459)	0.720	1.101 (0.495–2.448)	0.813
Baseline GAD-7								1.231 (1.187–1.276)	**<0.001**

Model 1 is the unadjusted model; Model 2 adjusts for demographic and family socio-economic factors; Model 3 further adjusts for health and behavioral factors based on Model 2; Model 4 further incorporates the corresponding baseline symptom levels based on Model 3. Missing covariates were handled by multiple imputation (full conditional specification method, *m* = 20), and the imputation results were combined for inference. Bold values indicate *P* < 0.05.

**Table 3:** Longitudinal association between baseline sleep-related problems and follow-up positive depressive symptoms (*n* = 1,529).

Variable	Classification	Model 1		Model 2		Model 3		Model 4	
		OR (95% CI)	*p*	OR (95% CI)	*p*	OR (95% CI)	*p*	OR (95% CI)	*p*
Baseline PSQI-5		1.325 (1.247–1.408)	**<0.001**	1.319 (1.239–1.405)	**<0.001**	1.320 (1.239–1.406)	**<0.001**	1.097 (1.019–1.180)	**0.013**
Sex (male = 0)	Female			0.836 (0.634–1.103)	0.205	0.845 (0.638–1.120)	0.242	0.850 (0.624–1.157)	0.301
School (School A = 0)	School B			1.385 (0.939–2.042)	0.101	1.388 (0.942–2.047)	0.097	1.335 (0.869–2.053)	0.187
	School C			0.929 (0.560–1.540)	0.775	0.936 (0.564–1.552)	0.798	0.939 (0.539–1.636)	0.824
Grade (Grade 1 = 0)	Grade 2			0.766 (0.536–1.095)	0.143	0.769 (0.534–1.106)	0.156	0.812 (0.550–1.199)	0.295
	Grade 3			0.645 (0.452–0.922)	**0.016**	0.644 (0.434–0.955)	**0.029**	0.587 (0.380–0.907)	**0.016**
Residence (urban = 0)	Rural			1.399 (0.716–2.735)	0.326	1.380 (0.703–2.707)	0.349	1.617 (0.781–3.348)	0.195
Parental divorce (no = 0)	Yes			0.859 (0.572–1.289)	0.461	0.848 (0.565–1.272)	0.425	0.815 (0.524–1.269)	0.365
Father/mother deceased (no = 0)	Yes			0.687 (0.135–3.489)	0.648	0.688 (0.130–3.647)	0.658	0.672 (0.120–3.761)	0.650
Family structure (two-parent family = 0)	Single mother family			1.446 (0.884–2.367)	0.142	1.463 (0.894–2.395)	0.130	1.236 (0.706–2.164)	0.458
	Single father family			2.126 (0.927–4.877)	0.075	2.166 (0.935–5.016)	0.071	1.750 (0.717–4.272)	0.219
	Both parents absent			2.390 (1.015–5.628)	**0.046**	2.408 (1.019–5.692)	**0.045**	2.470 (0.933–6.540)	0.069
Only child (no = 0)	Yes			0.878 (0.648–1.188)	0.398	0.884 (0.652–1.198)	0.427	0.904 (0.646–1.265)	0.557
Father’s education level (primary school or below = 0)	Junior high school			1.402 (0.437–4.499)	0.569	1.405 (0.439–4.499)	0.565	1.468 (0.425–5.073)	0.542
	High school or technical secondary school			1.099 (0.342–3.525)	0.874	1.111 (0.345–3.575)	0.859	1.293 (0.371–4.511)	0.686
	Bachelor or associate degree			1.219 (0.377–3.943)	0.740	1.232 (0.380–3.993)	0.727	1.460 (0.410–5.203)	0.558
	Master’s degree or above			1.259 (0.330–4.804)	0.736	1.274 (0.333–4.870)	0.723	1.926 (0.455–8.153)	0.372
Mother’s education level (primary school or below = 0)	Junior high school			0.637 (0.149–2.728)	0.542	0.655 (0.153–2.799)	0.567	0.827 (0.193–3.541)	0.798
	High school or technical secondary school			0.722 (0.174–3.003)	0.654	0.735 (0.177–3.050)	0.671	1.096 (0.258–4.651)	0.901
	Bachelor or associate degree			0.564 (0.133–2.386)	0.435	0.577 (0.136–2.442)	0.454	0.751 (0.173–3.250)	0.701
	Master’s degree or above			0.491 (0.092–2.621)	0.404	0.504 (0.094–2.692)	0.422	0.670 (0.123–3.657)	0.643
Monthly household income per capita (yuan) (<2000 = 0)	2000–4,000			0.706 (0.311–1.601)	0.404	0.673 (0.294–1.536)	0.346	0.767 (0.317–1.855)	0.556
	4,000–6,000			0.739 (0.317–1.724)	0.483	0.710 (0.303–1.665)	0.430	0.737 (0.296–1.836)	0.512
	6,000–8,000			1.106 (0.485–2.523)	0.810	1.070 (0.466–2.454)	0.874	1.219 (0.499–2.979)	0.664
	8,000–10,000			0.641 (0.273–1.504)	0.307	0.615 (0.261–1.452)	0.267	0.715 (0.286–1.788)	0.473
	>10,000			1.059 (0.470–2.386)	0.890	1.025 (0.453–2.320)	0.952	1.161 (0.484–2.784)	0.738
Chronic disease (no = 0)	Yes					0.448 (0.130–1.546)	0.204	0.558 (0.157–1.986)	0.368
Weight (kg)						1.001 (0.978–1.025)	0.915	0.991 (0.966–1.017)	0.494
Prefer sleeping with light on (no = 0)	Yes					1.179 (0.780–1.782)	0.435	1.121 (0.713–1.763)	0.622
Eating after 9 p.m. (never = 0)	Occasionally			0.706 (0.311–1.601)	0.404	0.673 (0.294–1.536)	0.346	0.767 (0.317–1.855)	0.556
	Every day			0.739 (0.317–1.724)	0.483	0.710 (0.303–1.665)	0.430	0.737 (0.296–1.836)	0.512
Baseline CDI-S								1.371 (1.304–1.442)	**<0.001**

Model 1 is the unadjusted model; Model 2 adjusts for demographic and family socio-economic factors; Model 3 further adjusts for health and behavioral factors based on Model 2; Model 4 further incorporates the corresponding baseline symptom levels based on Model 3. Missing covariates were handled by multiple imputation (full conditional specification method, *m* = 20), and the imputation results were combined for inference. Bold values indicate *P* < 0.05.

**Table 4:** Longitudinal association between baseline sleep-related problems and follow-up coexisting anxiety and depressive symptoms (*n* = 1,529).

Variable	Classification	Model 1		Model 2		Model 3		Model 4	
		OR (95% CI)	*p*	OR (95% CI)	*p*	OR (95% CI)	*p*	OR (95% CI)	*p*
Baseline PSQI-5		1.429 (1.334–1.532)	**<0.001**	1.430 (1.331–1.537)	**<0.001**	1.431 (1.331–1.538)	**<0.001**	1.146 (1.049–1.251)	**0.003**
Sex (male = 0)	Female			0.950 (0.690–1.307)	0.752	0.947 (0.684–1.311)	0.742	0.938 (0.661–1.331)	0.721
School (School A = 0)	School B			1.026 (0.661–1.591)	0.910	1.027 (0.663–1.592)	0.905	0.972 (0.603–1.569)	0.909
	School C			0.778 (0.440–1.375)	0.388	0.780 (0.441–1.381)	0.394	0.787 (0.425–1.458)	0.447
Grade (Grade 1 = 0)	Grade 2			0.862 (0.568–1.309)	0.487	0.863 (0.564–1.320)	0.496	0.985 (0.627–1.545)	0.946
	Grade 3			0.808 (0.534–1.222)	0.313	0.811 (0.513–1.283)	0.371	0.773 (0.470–1.272)	0.312
Residence (urban = 0)	Rural			1.597 (0.745–3.423)	0.228	1.583 (0.736–3.407)	0.239	1.897 (0.834–4.315)	0.127
Parental divorce (no = 0)	Yes			0.807 (0.506–1.288)	0.368	0.803 (0.504–1.281)	0.357	0.763 (0.457–1.272)	0.299
Father/mother deceased (no = 0)	Yes			0.800 (0.122–5.258)	0.815	0.797 (0.114–5.577)	0.817	0.852 (0.119–6.095)	0.873
Family structure (two-parent family = 0)	Single mother family			1.513 (0.867–2.638)	0.145	1.527 (0.875–2.663)	0.136	1.228 (0.657–2.296)	0.520
	Single father family			1.996 (0.779–5.117)	0.150	2.021 (0.787–5.192)	0.143	1.629 (0.613–4.326)	0.327
	Both parents absent			1.832 (0.687–4.886)	0.227	1.852 (0.693–4.948)	0.219	1.556 (0.497–4.868)	0.447
Only child (no = 0)	Yes			0.779 (0.548–1.107)	0.163	0.779 (0.548–1.108)	0.165	0.755 (0.516–1.105)	0.148
Father’s education level (primary school or below = 0)	Junior high school			1.642 (0.443–6.094)	0.457	1.647 (0.446–6.089)	0.453	1.770 (0.462–6.787)	0.404
	High school or technical secondary school			1.168 (0.315–4.326)	0.816	1.181 (0.319–4.378)	0.803	1.410 (0.363–5.472)	0.619
	Bachelor or associate degree			1.278 (0.334–4.891)	0.719	1.291 (0.337–4.938)	0.709	1.446 (0.355–5.891)	0.606
	Master’s degree or above			1.580 (0.341–7.319)	0.558	1.593 (0.344–7.379)	0.550	2.231 (0.454–10.955)	0.322
Mother’s education level (Primary school or below = 0)	Junior high school			0.797 (0.167–3.811)	0.776	0.815 (0.170–3.897)	0.797	0.721 (0.147–3.545)	0.687
	High school or technical secondary school			0.992 (0.211–4.657)	0.992	1.003 (0.214–4.700)	0.997	1.115 (0.226–5.507)	0.894
	Bachelor or associate degree			0.731 (0.151–3.527)	0.696	0.745 (0.154–3.606)	0.714	0.721 (0.141–3.686)	0.694
	Master’s degree or above			0.754 (0.124–4.583)	0.759	0.763 (0.126–4.628)	0.769	0.790 (0.126–4.970)	0.801
Monthly household income per capita (yuan) (<2000 = 0)	2000–4,000			0.777 (0.317–1.901)	0.580	0.760 (0.308–1.874)	0.551	0.804 (0.311–2.082)	0.654
	4,000–6,000			0.819 (0.326–2.059)	0.672	0.806 (0.319–2.036)	0.648	0.754 (0.284–1.997)	0.570
	6,000–8,000			1.152 (0.465–2.853)	0.759	1.140 (0.457–2.844)	0.778	1.204 (0.459–3.155)	0.706
	8,000–10,000			0.719 (0.279–1.855)	0.495	0.706 (0.272–1.832)	0.474	0.776 (0.284–2.115)	0.620
	>10,000			1.025 (0.418–2.515)	0.956	1.014 (0.411–2.501)	0.976	1.032 (0.400–2.664)	0.948
Chronic disease (no = 0)	Yes					0.699 (0.196–2.494)	0.581	0.948 (0.264–3.406)	0.934
Weight (kg)						1.000 (0.973–1.027)	0.991	0.991 (0.962–1.021)	0.556
Prefer sleeping with light on (no = 0)	Yes					1.224 (0.762–1.965)	0.403	1.123 (0.675–1.868)	0.656
Eating after 9 p.m. (never = 0)	Occasionally					0.949 (0.619–1.456)	0.811	0.944 (0.598–1.489)	0.803
	Every day					0.886 (0.331–2.374)	0.809	0.902 (0.323–2.522)	0.845
Baseline GAD-7								1.078 (1.029–1.130)	**0.002**
Baseline CDI-S								1.251 (1.180–1.326)	**<0.001**

Model 1 is the unadjusted model; Model 2 adjusts for demographic and family socio-economic factors; Model 3 further adjusts for health and behavioral factors based on Model 2; Model 4 further incorporates the corresponding baseline symptom levels based on Model 3. Missing covariates were handled by multiple imputation (full conditional specification method, *m* = 20), and the imputation results were combined for inference. Bold values indicate *P* < 0.05.

**Table 5:** Longitudinal association between baseline PSQI-5 quartiles and follow-up coexisting anxiety and depressive symptoms (*n* = 1,529).

Variable	Classification	Model 1		Model 2		Model 3		Model 4	
		OR (95% CI)	*p*	OR (95% CI)	*p*	OR (95% CI)	*p*	OR (95% CI)	*p*
Baseline PSQI-5	Q1	1.000	–	1.000	–	1.000	–	1.000	–
	Q2	1.889 (1.087–3.285)	**0.024**	1.849 (1.057–3.236)	**0.031**	1.849 (1.056–3.238)	**0.031**	1.496 (0.835–2.681)	0.176
	Q3	2.718 (1.657–4.459)	**<0.001**	2.624 (1.586–4.342)	**<0.001**	2.633 (1.590–4.361)	**0.001**	1.584 (0.927–2.707)	0.093
	Q4	7.781 (4.820–12.561)	**<0.001**	7.675 (4.696–12.545)	**<0.001**	7.700 (4.707–12.595)	**<0.001**	2.415 (1.370–4.258)	**0.002**
	Trend test	1.984 (1.705–2.309)	**<0.001**	1.976 (1.689–2.311)	**<0.001**	1.978 (1.691–2.315)	**<0.001**	1.311 (1.097–1.567)	**0.003**

Model 1 is the unadjusted model; Model 2 adjusts for demographic and family socio-economic factors; Model 3 further adjusts for health and behavioral factors based on Model 2; Model 4 further incorporates the corresponding baseline symptom levels based on Model 3. Missing covariates were handled by multiple imputation (full conditional specification method, *m* = 20), and the imputation results were combined for inference. Bold values indicate *P* < 0.05.

**Table 6:** Longitudinal association between change in sleep-related problems and follow-up coexisting anxiety and depressive symptoms (*n* = 1,529).

Variable	Classification	Model 1		Model 2		Model 3		Model 4	
		*β* (95% CI)	*p*	*β* (95% CI)	*p*	*β* (95% CI)	*p*	*β* (95% CI)	*p*
**∆PSQI**		1.521 (1.402–1.650)	**<0.001**	1.541 (1.416–1.678)	**<0.001**	1.544 (1.418–1.680)	**<0.001**	1.486 (1.359–1.626)	**<0.001**
Baseline PSQI-5		1.824 (1.666–1.997)	**<0.001**	1.854 (1.686–2.039)	**<0.001**	1.858 (1.689–2.044)	**<0.001**	1.499 (1.342–1.674)	**<0.001**
Sex (male = 0)	Female			1.041 (0.741–1.463)	0.815	1.043 (0.738–1.474)	0.811	1.032 (0.714–1.491)	0.868
Grade (grade 1 = 0)	Grade 2			0.855 (0.547–1.334)	0.490	0.855 (0.542–1.350)	0.501	0.978 (0.607–1.577)	0.929
	Grade 3			0.797 (0.511–1.243)	0.317	0.782 (0.476–1.285)	0.332	0.785 (0.461–1.334)	0.370
Residence (urban = 0)	Rural			1.784 (0.787–4.047)	0.165	1.783 (0.783–4.063)	0.168	1.891 (0.794–4.501)	0.149
Family structure (two-parent family = 0)	Single mother family			1.428 (0.785–2.599)	0.243	1.456 (0.799–2.652)	0.220	1.226 (0.628–2.392)	0.551
	Single father family			2.236 (0.813–6.149)	0.118	2.282 (0.827–6.297)	0.111	1.848 (0.656–5.210)	0.245
	Both parents absent			2.693 (0.915–7.925)	0.072	2.739 (0.927–8.098)	0.068	2.452 (0.781–7.699)	0.125
Baseline GAD-7								1.066 (1.014–1.121)	**0.012**
Baseline CDI-S								1.230 (1.157–1.308)	**<0.001**

Complete results for all covariates in this model are provided in Supplementary Table S1. Model 1 is the unadjusted model; Model 2 adjusts for demographic and family socio-economic factors; Model 3 further adjusts for health and behavioral factors based on Model 2; Model 4 further incorporates the corresponding baseline symptom levels based on Model 3. Missing covariates were handled by multiple imputation (full conditional specification method, *m* = 20), and the imputation results were combined for inference. Bold values indicate *P* < 0.05.

**Table 7:** Multiple linear regression of sleep-related problem changes and follow-up anxiety symptoms (*n* = 1,529).

Variable	Classification	Model 1		Model 2		Model 3	
		*β* (95% CI)	*p*	*β* (95% CI)	*p*	*β* (95% CI)	*p*
∆PSQI		0.893 (0.806–0.981)	**<0.001**	0.896 (0.807–0.984)	**<0.001**	0.896 (0.808–0.985)	**<0.001**
Baseline PSQI-5		0.775 (0.671–0.878)	**<0.001**	0.766 (0.661–0.871)	**<0.001**	0.765 (0.660–0.871)	**<0.001**
Baseline GAD-7		0.348 (0.303–0.393)	**<0.001**	0.344 (0.298–0.389)	**<0.001**	0.344 (0.298–0.390)	**<0.001**
Sex (male = 0)	Female			−0.165 (−0.488–0.159)	0.318	−0.171 (−0.501–0.159)	0.309
Grade (grade 1 = 0)	Grade 2			−0.149 (−0.584–0.285)	0.500	−0.111 (−0.553–0.331)	0.622
	Grade 3			−0.228 (−0.653–0.197)	0.293	−0.154 (−0.620–0.312)	0.516
Residence (urban = 0)	Rural			0.112 (−0.709–0.933)	0.790	0.141 (−0.684–0.965)	0.738
Family structure (two-parent family = 0)	Single mother family			0.420 (−0.185–1.025)	0.174	0.402 (−0.205–1.009)	0.194
	Single father family			0.467 (−0.553–1.488)	0.369	0.454 (−0.574–1.483)	0.386
	Both parents absent			0.958 (−0.225–2.141)	0.112	0.886 (−0.296–2.068)	0.142
Only child (no = 0)	Yes			−0.503 (−0.858 to −0.147)	**0.006**	−0.506 (−0.863 to −0.149)	**0.005**

Complete results for all covariates in this model are provided in Supplementary Table S2. Model 1 adjusted for ∆PSQI, baseline PSQI-5, and corresponding baseline symptom levels; Model 2 further adjusted for demographic and family socioeconomic factors based on Model 1; Model 3 further adjusted for health and behavioral factors based on Model 2. Missing covariates were handled by multiple imputation (full conditional specification method, *m* = 20), and the imputation results were combined for inference. Bold values indicate *P* < 0.05.

**Table 8:** Multiple linear regression of sleep-related problem changes and follow-up depressive symptoms (*n* = 1,529).

Variable	Classification	Model 1		Model 2		Model 3	
		*β* (95% CI)	*p*	*β* (95% CI)	*p*	*β* (95% CI)	*p*
∆PSQI		0.349 (0.284–0.414)	**<0.001**	0.346 (0.279–0.414)	**<0.001**	0.348 (0.280–0.415)	**<0.001**
Baseline PSQI-5		0.323 (0.251–0.394)	**<0.001**	0.306 (0.231–0.382)	**<0.001**	0.305 (0.230–0.381)	**<0.001**
Baseline CDI-S		0.451 (0.410–0.492)	**<0.001**	0.441 (0.397–0.484)	**<0.001**	0.440 (0.397–0.484)	**<0.001**
Sex (male = 0)	Female			0.008 (−0.239–0.255)	0.949	0.008 (−0.244–0.259)	0.953
Grade (grade 1 = 0)	Grade 2			−0.331 (−0.664–0.001)	0.051	−0.291 (−0.628–0.046)	0.090
	Grade 3			−0.645 (−0.970 to −0.319)	**<0.001**	−0.554 (−0.909 to −0.200)	**0.002**
Residence (urban = 0)	Rural			−0.037 (−0.669–0.595)	0.909	−0.044 (−0.677–0.589)	0.892
Family structure (two-parent family = 0)	Single mother family			0.343 (−0.106–0.791)	0.135	0.345 (−0.104–0.794)	0.132
	Single father family			0.592 (−0.202–1.386)	0.144	0.601 (−0.195–1.397)	0.139
	Both parents absent			0.998 (0.108–1.888)	**0.028**	0.961 (0.068–1.853)	**0.035**
Only child (no = 0)	Yes			−0.190 (−0.448–0.067)	0.148	−0.179 (−0.437–0.079)	0.174

Complete results for all covariates in this model are provided in Supplementary Table S3. Model 1 adjusted for ∆PSQI, baseline PSQI-5, and corresponding baseline symptom levels; Model 2 further adjusted for demographic and family socioeconomic factors based on Model 1; Model 3 further adjusted for health and behavioral factors based on Model 2. Missing covariates were handled by multiple imputation (full conditional specification method, *m* = 20), and the imputation results were combined for inference. Bold values indicate *P* < 0.05.

**Table 9:** Multiple linear regression of sleep-related problem changes and anxiety symptom changes (*n* = 1,529).

Variable	Classification	Model 1		Model 2		Model 3	
		*β* (95% CI)	*p*	*β* (95% CI)	*p*	*β* (95% CI)	*p*
∆PSQI		0.893 (0.806–0.981)	**<0.001**	0.885 (0.793–0.977)	**<0.001**	0.886 (0.793–0.978)	**<0.001**
Baseline PSQI-5		0.775 (0.671–0.878)	**<0.001**	0.754 (0.644–0.863)	**<0.001**	0.753 (0.643–0.862)	**<0.001**
Baseline GAD-7		−0.652 (−0.697--0.607)	**<0.001**	−0.646 (−0.694--0.597)	**<0.001**	−0.645 (−0.693--0.597)	**<0.001**
Sex (male = 0)	Female			−0.187 (−0.524–0.150)	0.277	−0.186 (−0.529–0.157)	0.289
Grade (grade 1 = 0)	Grade 2			−0.216 (−0.671–0.238)	0.350	−0.187 (−0.648–0.274)	0.426
	Grade 3			−0.304 (−0.748–0.141)	0.180	−0.250 (−0.735–0.234)	0.311
Residence (urban = 0)	Rural			0.160 (−0.701–1.022)	0.715	0.188 (−0.676–1.051)	0.670
Family structure (two-parent family = 0)	Single mother family			0.397 (−0.217–1.010)	0.205	0.382 (−0.232–0.996)	0.223
	Single father family			0.673 (−0.408–1.755)	0.223	0.647 (−0.437–1.732)	0.242
	Both parents absent			1.154 (−0.060–2.368)	0.062	1.081 (−0.137–2.300)	0.082
Only child (no = 0)	Yes			−0.555 (−0.907--0.204)	**0.002**	−0.559 (−0.911 to −0.207)	**0.002**

Complete results for all covariates in this model are provided in Supplementary Table S4. Model 1 adjusted for ∆PSQI, baseline PSQI-5, and corresponding baseline symptom levels; Model 2 further adjusted for demographic and family socioeconomic factors based on Model 1; Model 3 further adjusted for health and behavioral factors based on Model 2. Missing covariates were handled by multiple imputation (full conditional specification method, *m* = 20), and the imputation results were combined for inference. Bold values indicate *P* < 0.05.

**Table 10:** Multiple linear regression of sleep-related problem changes and depressive symptom changes (*n* = 1,529).

Variable	Classification	Model 1		Model 2		Model 3	
		*β* (95% CI)	*p*	*β* (95% CI)	*p*	*β* (95% CI)	*p*
∆PSQI		0.349 (0.284–0.414)	**<0.001**	0.358 (0.293–0.423)	**<0.001**	0.359 (0.294–0.424)	**<0.001**
Baseline PSQI-5		0.323 (0.251–0.394)	**<0.001**	0.325 (0.253–0.398)	**<0.001**	0.324 (0.251–0.397)	**<0.001**
Baseline CDI-S		−0.549 (−0.590--0.508)	**<0.001**	−0.560 (−0.601 to −0.518)	**<0.001**	−0.559 (−0.601 to −0.518)	**<0.001**
Sex (male = 0)	Female			0.092 (−0.146–0.331)	0.448	0.086 (−0.156–0.328)	0.487
Grade (grade 1 = 0)	Grade 2			−0.297 (−0.616–0.022)	0.068	−0.245 (−0.570–0.079)	0.138
	Grade 3			−0.593 (−0.905 to −0.281)	**<0.001**	−0.478 (−0.820 to −0.136)	**0.006**
Residence (urban = 0)	Rural			−0.040 (−0.640–0.560)	0.896	−0.046 (−0.646–0.554)	0.880
Family structure (two-parent family = 0)	Single mother family			0.324 (−0.138–0.786)	0.169	0.328 (−0.134–0.790)	0.165
	Single father family			0.541 (−0.257–1.339)	0.183	0.550 (−0.251–1.350)	0.178
	Both parents absent			0.907 (0.030–1.784)	**0.043**	0.862 (−0.019–1.743)	0.055
Only child (no = 0)	Yes			−0.167 (−0.427–0.092)	0.207	−0.156 (−0.415–0.104)	0.240

Complete results for all covariates in this model are provided in Supplementary Table S5. Model 1 adjusted for ∆PSQI, baseline PSQI-5, and corresponding baseline symptom levels; Model 2 further adjusted for demographic and family socioeconomic factors based on Model 1; Model 3 further adjusted for health and behavioral factors based on Model 2. Missing covariates were handled by multiple imputation (full conditional specification method, *m* = 20), and the imputation results were combined for inference. Bold values indicate *P* < 0.05.

**Table 11:** Baseline characteristics of participants with and without missing covariate data.

Variable	Complete data group (*n* = 1,405)		Missing data group (*n* = 124)		*t*/*χ^2^*	*p*
	Mean (SD)	*n* (%)	Mean (SD)	*n* (%)		
Baseline PSQI-5 total scores	4.79 (2.12)		4.65 (2.11)		0.707	0.480
Baseline GAD-7 total scores	4.48(4.17)		4.53 (4.57)		−0.121	0.904
Baseline CDI-S total scores	4.56 (3.18)		4.98 (3.61)		−1.381	0.167
Sociodemographic characteristics						
Age (years)	7.99 (1.03)		7.92 (1.12)		0.547	0.585
Sex					0.071	0.790
Male		720 (51.2)		62 (50.0)		
Female		685 (48.8)		62 (50.0)		

Complete results for all covariates in this model are provided in Supplementary Table S6. Data are presented as mean (SD) or *n* (%). *p* values: *t*-test for continuous variables, *χ*^2^ test for categorical variables (complete vs. missing groups). Percentages were based on non-missing cases.

**Table 12:** Sensitivity analysis of the associations between baseline sleep-related problems and follow-up emotional outcomes (n = 1,405).

Outcome	Model 1		Model 2		Model 3		Model 4	
	OR (95% CI)	*p*	OR (95% CI)	*p*	OR (95% CI)	*p*	OR (95% CI)	*p*
Follow-up positive anxiety symptoms	1.379 (1.302–1.462)	**<0.001**	1.362 (1.283–1.444)	**<0.001**	1.361 (1.283–1.444)	**<0.001**	1.215 (1.140–1.295)	**<0.001**
Follow-up positive depressive symptoms	1.327 (1.245–1.414)	**<0.001**	1.316 (1.232–1.407)	**<0.001**	1.318 (1.233–1.408)	**<0.001**	1.170 (1.088–1.259)	**<0.001**
Follow-up coexisting anxiety and depressive symptoms	1.432 (1.332–1.540)	**<0.001**	1.428 (1.324–1.540)	**<0.001**	1.429 (1.331–1.541)	**<0.001**	1.147 (1.046–1.258)	**0.004**

Complete results for all covariates in this model are provided in Supplementary Tables S7–S9. Model 1 is the unadjusted model; Model 2 adjusts for demographic and family socio-economic factors; Model 3 further adjusts for health and behavioral factors based on Model 2; Model 4 further incorporates the corresponding baseline symptom levels based on Model 3. Bold values indicate *P* < 0.05.

### 2.4 Quality control

Quality control was implemented throughout the study. A pilot survey was conducted prior to the formal investigation to optimize the questionnaire and field procedures. All investigators received standardized training before school visits. After questionnaire collection, double-entry verification was performed using EpiData, and logical error checks were conducted to ensure data accuracy and completeness.

## 3 Results

### 3.1 Basic characteristics of the study subjects

A total of 1,620 school-aged children (839 boys, 51.8%; 781 girls, 48.2%) was included in the baseline survey, and 1,744 children (895 boys, 51.3%; 849 girls, 48.7%) were included in the follow-up survey. The median age (interquartile range) for the two surveys was 8.00 (2.00) and 8.00 (1.00) years, and the median weight (interquartile range) were 26.40 (7.50) and 28.80 (9.10) kilograms, respectively.

The median (interquartile range) of the overall PSQI-5 scale in both the baseline and follow-up surveys was 4.00 (3.00). The detection rates of anxiety, depression, and the coexistence of anxiety and depression symptoms at baseline were 38.9%, 22.3%, and 16.5%; and at follow-up, they were 35.8%, 19.1%, and 13.1%, respectively. Distributions of other sociodemographic and family characteristics are presented in Table 1.

### 3.2 Short-term longitudinal association between baseline sleep-related issues and follow-up emotional outcomes

After adjusting for demographic, family, health-behavior factors, and corresponding baseline symptom levels, baseline PSQI-5 remained significantly associated with screening-positive anxiety (OR = 1.144, 95% CI: 1.071–1.222, *p* < 0.001) and depression (OR = 1.097, 95% CI: 1.019–1.180, *p* = 0.013) (Tables 2, 3).

For co-occurring anxiety and depression at follow-up, each 1-point increase in baseline PSQI-5 was associated with an OR of 1.146 (95% CI: 1.049–1.251, *p* = 0.003) in the fully adjusted model. When baseline PSQI-5 was categorized into quartiles (with Q1 as reference), the Q4 group remained significantly associated with co-occurring anxiety and depression at follow-up after full adjustment (Tables 4, 5).

### 3.3 Association between changes in sleep-related issues and follow-up emotional outcomes and symptom changes

Among the 1,529 children, ∆PSQI was positively associated with co-occurring anxiety and depression at follow-up. After adjusting for baseline PSQI-5, baseline GAD-7, baseline CDI-S, and other covariates, each 1-point increase in ΔPSQI was associated with an OR of 1.486 (95% CI: 1.359–1.626, *p* < 0.001) for co-occurrence; baseline PSQI-5 was also independently associated (OR = 1.499, 95% CI: 1.342–1.674, *p* < 0.001).

In the linear regression model, ΔPSQI, baseline PSQI-5, and corresponding baseline symptom levels were entered simultaneously and further adjusted for demographic, family socioeconomic, health, and behavior-related covariates. The results showed that ∆PSQI was positively associated with the follow-up GAD-7 total score, the follow-up CDI-S total score, ∆GAD, and ∆CDI (Tables 7–10).

### 3.4 Sensitivity analysis

Among the 1,529 children who completed the two surveys, a total of 1,405 (91.9%) had complete covariate data, and 124 (8.1%) had at least one missing covariate. The highest missing rate was for the only-child variable (*n* = 124); missing counts for other covariates (residence, parental divorce, father/mother deceased, family structure, father’s education level, mother’s education level, monthly household income per capita, prefer sleeping with light on and eating after 9 p.m.) were 122. Baseline characteristics were similar between the complete data group and missing data group (Table 11), with no significant differences in age, sex, baseline PSQI-5, GAD-7, or CDI-S scores (all *p* > 0.05), suggesting that missingness was likely random.

Among the 1,405 children, after adjusting for covariates and corresponding baseline symptom levels, baseline PSQI-5 remained positively associated with follow-up anxiety, depression and the coexistence of anxiety and depression (OR = 1.215, 1.170, and 1.147, *p* < 0.05) (Table 12). The associations between ΔPSQI and follow-up emotional outcomes and symptom changes also remained stable (*p* < 0.001), with full results presented in Supplementary Tables S10–S15. Overall, the sensitivity analysis confirmed the robustness of the main findings.

## 4 Discussion

Among the 1,529 children, higher baseline sleep-related problems were associated with a greater likelihood of screening-positive anxiety, depression, and their co-occurrence at follow-up. Further analysis revealed that the worsening of sleep-related problems during the observation period was associated with higher levels of emotional symptoms at the follow-up and changes in symptoms. The following discussion focuses on the epidemiological significance of these findings and their possible interpretations.

### 4.1 Short-term longitudinal association between baseline sleep-related issues and subsequent emotional outcomes and their differences

In the short-term longitudinal analysis, baseline sleep-related problems were associated with subsequent screening-positive anxiety, depression, and their co-occurrence, with the association being more stable for anxiety and co-occurring symptoms. The overall direction of previous research reports is consistent with the results of this study. A Canadian population study found cross-sectional and longitudinal associations between sleep difficulties and internalizing problems in children and adolescents (29). The study of American elementary school children also found that greater sleep disturbance and impairment predicted worsening emotional-behavioral problems over time (30).

After adjusting for sociodemographic, family, health-behavior factors, and corresponding baseline symptom levels, baseline sleep-related problems remained significantly associated with subsequent anxiety and co-occurring symptoms, whereas the association with subsequent depression weakened. This suggests that the short-term longitudinal relationship between sleep problems and different internalizing outcomes is not uniform in primary school children, with the association being more robust for anxiety and co-occurring symptoms. Similar phenomena have also been observed in previous studies, where persistent sleep problems in childhood are associated with anxiety disorders and depressive disorders in adulthood, but the strength of the association between different outcomes is not completely consistent (31). Sleep problems in the childhood and adolescence stages could also predict subsequent generalized anxiety and depressive manifestations (32). On the other hand, twin studies in middle childhood showed that sleep problems at age 8 could predict depressive symptoms at age 10 (33). Childhood sleep trajectory studies also indicated that prolonged sleep latency over the long term is associated with higher depressive symptoms in adolescence (34).

Developmentally, our participants were primarily in grades 1–3. The depressive-related manifestations at this age stage are not completely consistent with those in adolescence and adulthood. Clinical studies have shown that in addition to low mood, children and adolescents with depression often exhibit irritability, unstable emotions, physical discomfort, decreased interest, and decline in school performance (35, 36). Moreover, depressive symptoms in children and adolescents often co-occur with anxiety and somatic complaints, with considerable symptom overlap (37), and irritability itself is a core feature of childhood depression (38). When baseline CDI-S was included in the model, the residual association between sleep problems and subsequent depression weakened, possibly due to the temporal stability of baseline depressive symptoms and symptom overlap. In contrast, the association between baseline sleep problems and subsequent anxiety and co-occurrence persisted more strongly, consistent with the link between hyperarousal and sleep-onset difficulties. Previous studies have shown that anxiety sensitivity remains associated with longer sleep latency in anxious children and adolescents even after controlling for anxiety severity, depression, and age (39), which aligns with our findings.

After controlling for baseline GAD-7 and CDI-S total scores, baseline PSQI-5 remained significantly associated with subsequent anxiety-depression co-occurrence, suggesting that sleep problems are not merely an additional feature of a single symptom dimension but are also linked to a broader internalizing burden. Existing studies have shown that anxiety and depression comorbidity often correspond to a higher symptom burden and more complex clinical manifestations (10).

This study does not directly evaluate the screening efficacy or clinical predictive performance of sleep indicators, so its practical significance lies primarily in providing supplementary clues for school-based mental health surveillance, rather than supporting independent screening tools.

### 4.2 The association between sleep changes, related sleep dimensions and co-occurrence of anxiety and depression symptoms

This study further revealed that after including ∆PSQI and baseline PSQI-5 simultaneously in the model, both were statistically associated with the co-occurrence of follow-up anxiety and depression symptoms. This indicates that the burden of emotional symptoms at follow-up is not only related to the severity of sleep-related problems at baseline, but also to the changes in sleep-related problems during the observation period. Previous studies suggest that compared with a single sleep level, persistent deviation from one‘s usual sleep pattern may provide additional information. Poor sleep regularity in adolescents is associated with lower sleep efficiency, longer sleep onset latency, and increased depressive symptoms over time (40), whereas higher regularity is linked to longer sleep duration, better sleep quality, and lower overall psychopathological burden (41). Although these findings are primarily from adolescent populations, their overall direction aligns with our results, suggesting an association between sleep changes and psychological outcomes.

From a methodological standpoint, change scores reflect intra-individual deviations from one’s usual sleep state more directly than a single measurement. In school-aged children, short-term sleep deterioration may correspond to changes in family routines, academic pressure, peer relationships, or electronic media use. Associations among sleep duration, screen time, and internalizing problems have been reported (42). Moreover, the relationship between electronic device use and sleep is not unidirectional (43–45). Although we did not include screen use, physical activity, academic stress, or parent–child relationships in our models, the observed synchronization between sleep changes and co-occurring symptom burden is consistent with existing evidence on lifestyle disruption and circadian rhythm disturbances. Since ∆PSQI consists of both baseline and follow-up measurements, and the follow-up outcome is located at the end of the same observation interval, this part of the result is more suitable as evidence of the concomitant association between sleep changes and emotional outcomes within the same observation interval.

Dimension-specific analyses showed that subjective sleep quality and sleep onset latency maintained relatively stable associations with subsequent co-occurrence, whereas sleep disturbances, hypnotic medication use, and daytime dysfunction showed attenuated or non-significant associations after adjusting for baseline emotional symptoms. It suggested that phenotypes closer to core nocturnal sleep processes have more stable relationships with later internalizing burden in primary-school children. Previous studies have shown that anxiety and depression-related sleep disturbances manifest not only as insufficient sleep duration, but also as difficulty in sleep initiation, impaired sleep continuity, and decreased rhythm consistency (46). When mood disorders in children and adolescents occurred simultaneously in children and adolescents, it usually indicated a higher risk and a greater burden of symptoms (47, 48). In this study, there was a past correlation between subjective sleep and the time of falling asleep, and this correlation was consistent with the subsequent occurrence of anxiety and depression symptoms. It was consistent with the above-mentioned evidence.

Although the observed odds ratios were modest (1.1–1.2 range), the cumulative population-level effect may be meaningful for screening purposes. Previous studies have shown that school environmental factors are related to the sleep health status of children and adolescents, and schools are also important venues for promoting sleep health (16). In the school scenario, mental health identification typically relies on multiple information sources—classroom performance, behavioral changes, peer relationships, attendance, and parental feedback. For children with poor subjective sleep quality or marked difficulty falling asleep, integrating sleep information with teacher observations, home-school communication, and routine screening results may facilitate a more comprehensive understanding of their emotional and behavioral changes. At the same time, school sleep interventions have certain coverage advantages, but single and short-term sleep education has limited effects. More multi-component intervention strategies that involve collaboration between schools and families are needed (49). Based on the results of this study, sleep-related issues are more suitable to be used as supplementary information in school-based mental health observations and home-school collaborative support.

### 4.3 Methodological considerations and generalizability

Several methodological considerations should inform the interpretation of our findings.

First, the PSQI-5 showed modest internal consistency (Cronbach’s *α*: 0.495–0.540). Previous studies have reported similarly low α values across different populations, ranging from 0.38 to 0.67 (50, 51). The lower reliability may increase the random error in exposure measurement, which typically attenuates rather than inflates the estimated associations. Nevertheless, after adjusting for confounding factors and baseline emotional levels, the baseline PSQI-5 still had a significant association with various follow-up emotional outcomes, which provided empirical support for its effectiveness in this study population. In summary, our results should be regarded as preliminary epidemiological evidence based on this research indicator, rather than the final conclusion of a fully validated pediatric sleep measurement tool. Its interpretation should be maintained with appropriate caution.

Second, the sample was drawn from three primary schools in a certain urban area of Chongqing, which was relatively concentrated in origin. Moreover, Chongqing, as an important central city in Southwest China, has a leading economic development level in the country, and its urban schools usually have better conditions for running schools, more regular work and rest management and more abundant health education resources, which may have an impact on children’s sleep patterns, daily behavior habits and mental health. Therefore, we should be cautious when extending the results of this study to rural schools, economically underdeveloped areas in the central and western regions or people with other cultural backgrounds. Further multi-center studies covering geographically and demographically diverse populations are needed to confirm the generalizability of the observed associations.

### 4.4 Innovation and limitations

This study has several strengths. Firstly, while much previous research has focused on adolescents, this study targeted younger school-aged children, adding to the evidence base on sleep-emotion associations in this under-studied age group. Secondly, by including co-occurring anxiety and depression as an outcome, we captured both single-symptom and higher-burden phenotypes, providing a more comprehensive picture of internalizing problems in school-aged children. Thirdly, the inclusion of a sleep change index (ΔPSQI) allowed us to examine dynamic associations between sleep trajectories and emotional outcomes. Fourthly, this study also further analyzed different sleep dimensions, thereby providing supplementary information for understanding the differences in the relationship between different sleep manifestations and emotional outcomes.

Several limitations should also be acknowledged. Firstly, anxiety and depression were assessed using screening scales (GAD-7 and CDI-S) rather than clinical diagnostic interviews; thus, our outcomes reflect screening-positive status, not clinical diagnoses. Secondly, although we adjusted for a range of covariates, residual confounding by unmeasured factors cannot be ruled out. Thirdly, while previous studies suggest bidirectional relationships between sleep and emotional symptoms, our analysis focused on the unidirectional effect of sleep on later emotional outcomes; reverse effects were not examined. If future research can obtain tracking data at more than three time points, it can further adopt cross-lagged panel models (CLPM) or random-intercept cross-lagged panel models (RI-CLPM), based on the separation of individual-level stable differences, to deeply reveal the interactive evolution mechanism between sleep and internalizing symptoms, thereby compensating for the shortcomings of this study.

## 5 Conclusion

Based on the survey data of school-aged children from three primary schools in a certain urban area of Chongqing City, this study analyzed the associations between PSQI-5-defined sleep-related problems and anxiety, depression, and their co-occurrence. The results showed that higher baseline sleep problems were associated with subsequent anxiety, depression, and co-occurring symptoms. During the observation period, the aggravation of sleep-related problems was related to greater co-occurrence burden and exacerbation of anxiety and depressive symptoms. Subjective sleep quality and sleep onset latency showed relatively stable associations with adverse emotional outcomes. Overall, these findings indicate that sleep-related problems may serve as supplementary information for monitoring children’s mental health in school and family settings. Given that this study used the PSQI-5 index, with a short follow-up and limited measurement time-points, conclusions should be interpreted only as epidemiological associations and observational references for school-based settings.

## Data Availability

The data that support the findings of this study are available from Research on the Mechanism of Early Onset of Adolescence in Children and Its Subsequent Health Effects but restrictions apply to the availability of these data, which were used under license for the current study, and so are not publicly available. Data are however available from the authors upon reasonable request and with permission of research on the Mechanism of Early Onset of Adolescence in Children and Its Subsequent Health Effects. Requests to access the datasets should be directed to Qin Liu, liuqin@cqmu.edu.cn.
