# Supplementary material for: Study on the association between sleep quality and anxiety–depression symptoms in school-aged children
Source: Front Public Health. 2026 Oct 20;14:1927475. doi: 10.3389/fpubh.2026.1927475 (PMC13727519; doi:10.3389/fpubh.2026.1927475)
Supplement: Supplementary file 1 [file Table_1.DOCX]

**Table S1. Longitudinal association between change in sleep-related problems and follow-up coexisting anxiety and depressive symptoms (n=1529)**

| **Variable** | **Classification** | **Model 1** | | **Model 2** | | **Model 3** | | | **Model 4** | |
| --- | --- | --- | --- | --- | --- | --- | --- | --- | --- | --- |
|  |  | ***OR* (95% *CI*)** | ***P*** | ***OR* (95% *CI*)** | ***P*** | ***OR* (95% *CI*)** | | ***P*** | ***OR* (95% *CI*)** | ***P*** |
| **∆PSQI** |  | 1.521 (1.402-1.650) | <0.001 | 1.541 (1.416-1.678) | <0.001 | 1.544 (1.418-1.680) | <0.001 | | 1.486 (1.359-1.626) | <0.001 |
| **Baseline PSQI-5** |  | 1.824 (1.666-1.997) | <0.001 | 1.854 (1.686-2.039) | <0.001 | 1.858 (1.689-2.044) | <0.001 | | 1.499 (1.342-1.674) | <0.001 |
| **Sex** (Male=0) | Female |  |  | 1.041 (0.741-1.463) | 0.815 | 1.043 (0.738-1.474) | 0.811 | | 1.032 (0.714-1.491) | 0.868 |
| **School** (School A=0) | School B |  |  | 1.194 (0.748-1.906) | 0.457 | 1.196 (0.750-1.908) | 0.453 | | 1.178 (0.710-1.957) | 0.526 |
|  | School C |  |  | 0.887 (0.483-1.627) | 0.698 | 0.883 (0.480-1.627) | 0.690 | | 0.939 (0.492-1.795) | 0.850 |
| **Grade** (Grade 1=0) | Grade 2 |  |  | 0.855 (0.547-1.334) | 0.490 | 0.855 (0.542-1.350) | 0.501 | | 0.978 (0.607-1.577) | 0.929 |
|  | Grade 3 |  |  | 0.797 (0.511-1.243) | 0.317 | 0.782 (0.476-1.285) | 0.332 | | 0.785 (0.461-1.334) | 0.370 |
| **Residence** (Urban=0) | Rural |  |  | 1.784 (0.787-4.047) | 0.165 | 1.783 (0.783-4.063) | 0.168 | | 1.891 (0.794-4.501) | 0.149 |
| **Parental divorce** (No=0) | Yes |  |  | 0.814 (0.494-1.339) | 0.417 | 0.808 (0.491-1.330) | 0.402 | | 0.752 (0.439-1.289) | 0.300 |
| **Father/mother deceased** (No=0) | Yes |  |  | 0.704 (0.096-5.133) | 0.726 | 0.707 (0.088-5.691) | 0.742 | | 0.787 (0.091-6.776) | 0.826 |
| **Family structure**  (Two-parent family=0) | Single mother family |  |  | 1.428 (0.785-2.599) | 0.243 | 1.456 (0.799-2.652) | 0.220 | | 1.226 (0.628-2.392) | 0.551 |
|  | Single father family |  |  | 2.236 (0.813-6.149) | 0.118 | 2.282 (0.827-6.297) | 0.111 | | 1.848 (0.656-5.210) | 0.245 |
|  | Both parents absent |  |  | 2.693 (0.915-7.925) | 0.072 | 2.739 (0.927-8.098) | 0.068 | | 2.452 (0.781-7.699) | 0.125 |
| **Only child** (No=0) | Yes |  |  | 0.824 (0.569-1.193) | 0.304 | 0.818 (0.564-1.186) | 0.289 | | 0.824 (0.555-1.223) | 0.336 |
| **Father's education level**  (Primary school or below=0) | Junior high school |  |  | 1.430 (0.361-5.671) | 0.609 | 1.418 (0.362-5.556) | 0.615 | | 1.636 (0.397-6.744) | 0.495 |
|  | High school or technical secondary school |  |  | 1.036 (0.261-4.117) | 0.959 | 1.044 (0.265-4.118) | 0.950 | | 1.217 (0.289-5.126) | 0.789 |
|  | Bachelor or associate degree |  |  | 1.216 (0.293-5.041) | 0.787 | 1.214 (0.296-4.981) | 0.787 | | 1.373 (0.308-6.118) | 0.677 |
|  | Master's degree or above |  |  | 1.401 (0.280-7.014) | 0.681 | 1.385 (0.279-6.879) | 0.689 | | 1.872 (0.347-10.107) | 0.465 |
| **Mother's education level**  (Primary school or below=0) | Junior high school |  |  | 0.933 (0.181-4.799) | 0.933 | 0.962 (0.187-4.945) | | 0.963 | 0.907 (0.159-5.159) | 0.912 |
|  | High school or technical secondary school |  |  | 1.335 (0.263-6.781) | 0.727 | 1.354 (0.268-6.845) | | 0.714 | 1.520 (0.266-8.701) | 0.638 |
|  | Bachelor or associate degree |  |  | 0.986 (0.189-5.146) | 0.986 | 1.014 (0.194-5.298) | | 0.986 | 1.003 (0.168-5.970) | 0.998 |
|  | Master's degree or above |  |  | 0.900 (0.137-5.905) | 0.912 | 0.928 (0.143-6.041) | | 0.938 | 0.957 (0.130-7.029) | 0.966 |
| **Monthly household income per capita (yuan)** (<2000=0) | 2000-4000 |  |  | 0.805 (0.320-2.025) | 0.645 | 0.788 (0.310-2.002) | | 0.617 | 0.809 (0.302-2.168) | 0.674 |
|  | 4000-6000 |  |  | 0.850 (0.328-2.205) | 0.738 | 0.837 (0.321-2.186) | | 0.716 | 0.759 (0.278-2.071) | 0.590 |
|  | 6000-8000 |  |  | 1.097 (0.427-2.822) | 0.847 | 1.078 (0.416-2.795) | | 0.877 | 1.066 (0.392-2.900) | 0.900 |
|  | 8000-10000 |  |  | 0.553 (0.203-1.504) | 0.246 | 0.536 (0.195-1.474) | | 0.227 | 0.578 (0.202-1.655) | 0.307 |
|  | >10000 |  |  | 1.006 (0.395-2.564) | 0.990 | 0.990 (0.386-2.540) | | 0.984 | 1.015 (0.378-2.724) | 0.976 |
| **Chronic disease** (No=0) | Yes |  |  |  |  | 0.618 (0.163-2.346) | | 0.480 | 0.776 (0.192-3.130) | 0.722 |
| **Weight (kg)** |  |  |  |  |  | 1.003 (0.974-1.032) | | 0.846 | 0.992 (0.961-1.024) | 0.621 |
| **Prefer sleeping with light on** (No=0) | Yes |  |  |  |  | 1.275 (0.771-2.108) | | 0.343 | 1.148 (0.667-1.978) | 0.618 |
| **Eating after 9 PM** (Never=0) | Occasionally |  |  |  |  | 0.901 (0.575-1.412) | | 0.649 | 0.913 (0.571-1.461) | 0.706 |
|  | Every day |  |  |  |  | 0.794 (0.266-2.372) | | 0.679 | 0.767 (0.250-2.355) | 0.643 |
| **Baseline GAD-7** |  |  |  |  |  |  | |  | 1.066 (1.014-1.121) | 0.012 |
| **Baseline CDI-S** |  |  |  |  |  |  | |  | 1.230 (1.157-1.308) | <0.001 |

*Note*: Model 1 is the unadjusted model; Model 2 adjusts for demographic and family socio-economic factors; Model 3 further adjusts for health and behavioral factors based on Model 2; Model 4 further incorporates the corresponding baseline symptom levels based on Model 3. Missing covariates were handled by multiple imputation (full conditional specification method, m=20), and the imputation results were combined for inference.
