# Supplementary material for: Study on the association between sleep quality and anxiety–depression symptoms in school-aged children
Source: Front Public Health. 2026 Oct 20;14:1927475. doi: 10.3389/fpubh.2026.1927475 (PMC13727519; doi:10.3389/fpubh.2026.1927475)
Supplement: Supplementary file 2 [file Table_10.DOCX]

**Table S10. Longitudinal association between baseline PSQI-5 quartiles and follow-up coexisting anxiety and depressive symptoms (n=1405)**

| **Variable** | **Classification** | **Model 1** | | **Model 2** | | **Model 3** | | **Model 4** | |
| --- | --- | --- | --- | --- | --- | --- | --- | --- | --- |
|  |  | ***OR* (95% *CI*)** | ***P*** | ***OR* (95% *CI*)** | ***P*** | ***OR* (95% *CI*)** | ***P*** | ***OR* (95% *CI*)** | ***P*** |
| **Baseline PSQI-5** | **Q1** | 1.000 | - | 1.000 | - | 1.000 | - | 1.000 | - |
|  | **Q2** | 1.450 (0.921-2.282) | 0.109 | 1.396 (0.880-2.213) | 0.157 | 1.397 (0.880-2.217) | 0.156 | 1.435 (0.709-2.866) | 0.173 |
|  | **Q3** | 1.978 (1.318-2.967) | <0.001 | 1.808(1.194-2.739) | 0.005 | 1.820 (1.200-2.759) | 0.005 | 1.387 (0.727-2.705) | 0.122 |
|  | **Q4** | 4.760 (3.180-7.126) | <0.001 | 4.437 (2.925-6.731) | <0.001 | 4.469 (2.942-6.787) | <0.001 | 3.415 (2.307-6.575) | 0.003 |
|  | **Trend test** | 1.673 (1.476-1.908) | <0.001 | 1.628 (1.420-1.866) | <0.001 | 1.632 (1.424-1.872) | <0.001 | 1.121 (1.007-1.287) | 0.005 |

*Note*: Model 1 is the unadjusted model; Model 2 adjusts for demographic and family socio-economic factors; Model 3 further adjusts for health and behavioral factors based on Model 2; Model 4 further incorporates the corresponding baseline symptom levels based on Model 3.
