# Supplementary material for: Study on the association between sleep quality and anxiety–depression symptoms in school-aged children
Source: Front Public Health. 2026 Oct 20;14:1927475. doi: 10.3389/fpubh.2026.1927475 (PMC13727519; doi:10.3389/fpubh.2026.1927475)
Supplement: Supplementary file 3 [file Table_11.DOCX]

**Table S11. Longitudinal association between change in sleep-related problems and follow-up coexisting anxiety and depressive symptoms (n=1405)**

| **Variable** | **Classification** | **Model 1** | | **Model 2** | | **Model 3** | | **Model 4** | |
| --- | --- | --- | --- | --- | --- | --- | --- | --- | --- |
|  |  | ***OR* (95% *CI*)** | ***P*** | ***OR* (95% *CI*)** | ***P*** | ***OR* (95% *CI*)** | ***P*** | ***OR* (95% *CI*)** | ***P*** |
| **∆PSQI** |  | 1.520 (1.395-1.656) | <0.001 | 1.543 (1.412-1.687) | <0.001 | 1.546 (1.414-1.690) | <0.001 | 1.485 (1.352-1.632) | <0.001 |
| **Baseline PSQI-5** |  | 1.816 (1.654-1.994) | <0.001 | 1.842 (1.669-2.033) | <0.001 | 1.847 (1.673-2.039) | <0.001 | 1.500 (1.337-1.682) | <0.001 |
| **Sex** (Male=0) | Female |  |  | 1.013 (0.708-1.448) | 0.944 | 1.018 (0.708-1.464) | 0.922 | 0.975 (0.663-1.432) | 0.897 |
| **School** (School A=0) | School B |  |  | 1.114 (0.687-1.806) | 0.662 | 1.117 (0.689-1.811) | 0.652 | 1.082 (0.643-1.820) | 0.767 |
|  | School C |  |  | 0.769 (0.397-1.488) | 0.435 | 0.764 (0.393-1.484) | 0.426 | 0.761 (0.379-1.531) | 0.444 |
| **Grade** (Grade 1=0) | Grade 2 |  |  | 0.826 (0.518-1.317) | 0.421 | 0.823 (0.510-1.327) | 0.424 | 0.930 (0.567-1.526) | 0.774 |
|  | Grade 3 |  |  | 0.728 (0.455-1.164) | 0.185 | 0.705 (0.419-1.187) | 0.188 | 0.685-(0.393-1.193) | 0.181 |
| **Residence** (Urban=0) | Rural |  |  | 1.982 (0.886-4.432) | 0.096 | 1.979 (0.882-4.442) | 0.098 | 2.232 (0.962-5.180) | 0.062 |
| **Parental divorce** (No=0) | Yes |  |  | 0.779 (0.476-1.276) | 0.322 | 0.772 (0.417-1.266) | 0.306 | 0.718 (0.421-1.226) | 0.225 |
| **Father/mother deceased** (No=0) | Yes |  |  | 0.723 (0.078-6.733) | 0.776 | 0.734 (0.079-6.812) | 0.786 | 0.786 (0.071-8.685) | 0.844 |
| **Family structure**  (Two-parent family=0) | Single mother family |  |  | 1.480 (0.814-2.694) | 0.199 | 1.509 (0.828-2.748) | 0.179 | 1.272 (0.659-2.454) | 0.473 |
|  | Single father family |  |  | 2.685 (1.026-7.026) | **0.044** | 2.743 (1.044-7.204) | **0.041** | 2.125 (0.769-5.875) | 0.146 |
|  | Both parents absent |  |  | 3.368 (1.138-9.996) | **0.028** | 3.422 (1.148-10.199) | **0.027** | 2.981 (0.935-9.501) | 0.065 |
| **Only child** (No=0) | Yes |  |  | 0.812 (0.557-1.185) | 0.280 | 0.804 (0.550-1.176) | 0.261 | 0.803 (0.535-1.204) | 0.288 |
| **Father's education level**  (Primary school or below=0) | Junior high school |  |  | 2.015 (0.336-12.080) | 0.443 | 1.977 (0.334-11.713) | 0.453 | 2.287 (0.372-14.051) | 0.372 |
|  | High school or technical secondary school |  |  | 1.456 (0.238-8.921) | 0.685 | 1.449 (0.238-8.813) | 0.687 | 1.681 (0.263-10.736) | 0.583 |
|  | Bachelor or associate degree |  |  | 1.728 (0.278-10.728) | 0.557 | 1.699 (0.276-10.468) | 0.568 | 1.900 (0.295-12.242) | 0.500 |
|  | Master's degree or above |  |  | 2.020 (0.269-15.155) | 0.494 | 1.986 (0.267-14.775) | 0.503 | 2.653 (0.337-20.903) | 0.354 |
| **Mother's education level**  (Primary school or below=0) | Junior high school |  |  | 0.724 (0.121-4.316) | 0.723 | 0.755 (0.129-4.917) | 0.755 | 0.711 (0.109-4.656) | 0.722 |
|  | High school or technical secondary school |  |  | 1.028 (0.172-6.139) | 0.976 | 1.057 (0.181-6.180) | 0.951 | 1.176 (0.178-7.782) | 0.866 |
|  | Bachelor or associate degree |  |  | 0.741 (0.123-4.486) | 0.744 | 0.775 (0.130-4.609) | 0.779 | 0.763 (0.114-5.112) | 0.780 |
|  | Master's degree or above |  |  | 0.633 (0.081-4.951) | 0.663 | 0.667 (0.087-5.121) | 0.697 | 0.684 (0.078-6.027) | 0.732 |
| **Monthly household income per capita (yuan)** (<2000=0) | 2000-4000 |  |  | 0.675 (0.255-1.786) | 0.429 | 0652 (0.244-1.745) | 0.395 | 0.688 (0.245-1.929) | 0.477 |
|  | 4000-6000 |  |  | 0.703 (0.257-1.921) | 0.493 | 0.685 (0.249-1.888) | 0.465 | 0.631 (0.218-1.825) | 0.396 |
|  | 6000-8000 |  |  | 0.917 (0.339-2.485) | 0.866 | 0.888 (0.325-2.430) | 0.818 | 0.891 (0.310-2.560) | 0.830 |
|  | 8000-10000 |  |  | 0.435 (0.152-1.244) | 0.121 | 0.416 (0.144-1.201) | 0.105 | 0.460 (0.152-1.390) | 0.169 |
|  | >10000 |  |  | 0.840 (0.312-2.263) | 0.730 | 0.817 (0.301-2.220) | 0.693 | 0.856 (0.302-2.429) | 0.770 |
| **Chronic disease** (No=0) | Yes |  |  |  |  | 0.618 (0.161-2.368) | 0.482 | 0.777 (0.191-3.157) | 0.724 |
| **Weight（kg）** |  |  |  |  |  | 1.005 (0.975-1.036) | 0.740 | 0.996 (0.964-1.029) | 0.825 |
| **Prefer sleeping with light on** (No=0) | Yes |  |  |  |  | 1.327 (0.802-2.195) | 0.270 | 1.174 (0.680-2.027) | 0.564 |
| **Eating after 9 PM** (Never=0) | Occasionally |  |  |  |  | 0.922 (0.585-1.456) | 0.729 | 0.934 (0.582-1.500) | 0.779 |
|  | Every day |  |  |  |  | 0.745 (0.242-2.300) | 0.609 | 0.714 (0.219-2.321) | 0.575 |
| **Baseline GAD-7** |  |  |  |  |  |  |  | 1.063 (1.008-1.120) | 0.024 |
| **Baseline CDI-S** |  |  |  |  |  |  |  | 1.223 (1.147-1.305) | <0.001 |

*Note*: Model 1 is the unadjusted model; Model 2 adjusts for demographic and family socio-economic factors; Model 3 further adjusts for health and behavioral factors based on Model 2; Model 4 further incorporates the corresponding baseline symptom levels based on Model 3.
