# Supplementary material for: Study on the association between sleep quality and anxiety–depression symptoms in school-aged children
Source: Front Public Health. 2026 Oct 20;14:1927475. doi: 10.3389/fpubh.2026.1927475 (PMC13727519; doi:10.3389/fpubh.2026.1927475)
Supplement: Supplementary file 6 [file Table_14.DOCX]

**Table S14. Multiple linear regression of sleep-related problem changes and anxiety symptom changes (n=1405)**

| **Variable** | **Classification** | **Model 1** | | **Model 2** | | **Model 3** | |
| --- | --- | --- | --- | --- | --- | --- | --- |
|  |  | ***β* (95% *CI*)** | ***P*** | ***β* (95% *CI*)** | ***P*** | ***β* (95% *CI*)** | ***P*** |
| **∆PSQI** |  | 0.881 (0.790-0.973) | <0.001 | 0.885 (0.792-0.977) | <0.001 | 0.886 (0.793-0.978) | <0.001 |
| **Baseline PSQI-5** |  | 0.764 (0.656-0.872) | <0.001 | 0.754 (0.644-0.863) | **<0.001** | 0.753 (0.643-0.862) | <0.001 |
| **Baseline GAD-7** |  | -0.640 (-0.688--0.592) | <0.001 | -0.646 (-0.694--0.597) | **<0.001** | -0.645 (-0.693--0.596) | <0.001 |
| **Sex** (Male=0) | Female |  |  | 0.187 (-0.151-0.525) | 0.278 | -0.186 (-0.158-0.529) | 0.289 |
| **School** (School A=0) | School B |  |  | 0.258 (-0.199-0.715) | 0.269 | 0.255 (-0.203-0.712) | 0.276 |
|  | School C |  |  | 0.165 (-0.456-0.786) | 0.602 | 0.181 (-0.441-0.803) | 0.568 |
| **Grade** (Grade 1=0) | Grade 2 |  |  | -0.216 (-0.671-0.238) | 0.350 | -0.187 (-0.649-0.274) | 0.426 |
|  | Grade 3 |  |  | -0.304 (-0.749-0.141) | 0.181 | -0.250 (-0.736-0.235) | 0.311 |
| **Residence** (Urban=0) | Rural |  |  | 0.160 (-0.702-1.023) | 0.715 | 0.188 (-0.676-1.052) | 0.670 |
| **Parental divorce** (No=0) | Yes |  |  | 0.335 (-0.117-0.787) | 0.146 | 0.353 (-0.100-0.806) | 0.127 |
| **Father/mother deceased** (No=0) | Yes |  |  | 0.692 (-1.343-2.726) | 0.505 | 0.641 (-1.395-2.678) | 0.537 |
| **Family structure**  (Two-parent family=0) | Single mother family |  |  | 0.397 (-0.217-1.011) | 0.205 | 0.382 (-0.233-0.997) | 0.223 |
|  | Single father family |  |  | 0.673 (-0.409-1.756) | 0.223 | 0.647 (-0.438-1.732) | 0.242 |
|  | Both parents absent |  |  | 1.154 (-0.061-2.369) | 0.063 | 1.081 (-0.139-2.301) | 0.082 |
| **Only child** (No=0) | Yes |  |  | -0.555 (-0.907--0.204) | 0.002 | -0.559 (-0.911--0.206) | 0.002 |
| **Father's education level**  (Primary school or below=0) | Junior high school |  |  | 0.087 (-1.596-1.771) | 0.919 | 0.118 (-1.568-1.804) | 0.891 |
|  | High school or technical secondary school |  |  | -0.413 (-2.105-1.279) | 0.632 | -0.389 (-2.084-1.307) | 0.653 |
|  | Bachelor or associate degree |  |  | -0.428 (-2.133-1.277) | 0.622 | -0.421 (-2.129-1.288) | 0.629 |
|  | Master's degree or above |  |  | -0.665 (-2.585-1.255) | 0.497 | -0.681 (-2.602-1.241) | 0.487 |
| **Mother's education level**  (Primary school or below=0) | Junior high school |  |  | -1.257 (-3.219-0.705) | 0.209 | -1.248 (-3.213-0.718) | 0.213 |
|  | High school or technical secondary school |  |  | -0.859 (-2.822-1.104) | 0.391 | -0.857 (-2.823-1.108) | 0.392 |
|  | Bachelor or associate degree |  |  | -0.969 (-2.942-1.005) | 0.336 | -0.945 (-2.923-1.032) | 0.348 |
|  | Master's degree or above |  |  | -0.763 (-2.978-1.452) | 0.499 | -0.755 (-2.972-1.462) | 0.504 |
| **Monthly household income per capita (yuan)** (<2000=0) | 2000-4000 |  |  | 0.269 (-0.773-1.312) | 0.612 | 0.292 (-0.756-1.340) | 0.585 |
|  | 4000-6000 |  |  | 0.060 (-1.006-1.126) | 0.912 | 0.080 (-0.989-1.149) | 0.883 |
|  | 6000-8000 |  |  | 0.177 (-0.899-1.253) | 0.747 | 0.203 (-0.877-1.283) | 0.712 |
|  | 8000-10000 |  |  | -0.236 (-1.314-0.843) | 0.668 | -0.205 (-1.287-0.877) | 0.710 |
|  | >10000 |  |  | 0.092 (-0.967-1.150) | 0.865 | 0.111 (-0.951-1.173) | 0.838 |
| **Chronic disease** (No=0) | Yes |  |  |  |  | -0.320 (-1.391-0.751) | 0.558 |
| **Weight（kg）** |  |  |  |  |  | -0.007 (-0.036-0.021) | 0.615 |
| **Prefer sleeping with light on** (No=0) | Yes |  |  |  |  | -0.346 (-0.842-0.150) | 0.171 |
| **Eating after 9 PM** (Never=0) | Occasionally |  |  |  |  | -0.177 (-0.609-0.255) | 0.422 |
|  | Every day |  |  |  |  | 0.067 (-1.007-1.140) | 0.903 |

*Note*: Model 1 adjusted for ∆PSQI, baseline PSQI-5, and corresponding baseline symptom levels; Model 2 further adjusted for demographic and family socioeconomic factors based on Model 1; Model 3 further adjusted for health and behavioral factors based on Model 2.
