# Supplementary material for: Study on the association between sleep quality and anxiety–depression symptoms in school-aged children
Source: Front Public Health. 2026 Oct 20;14:1927475. doi: 10.3389/fpubh.2026.1927475 (PMC13727519; doi:10.3389/fpubh.2026.1927475)
Supplement: Supplementary file 7 [file Table_15.DOCX]

**Table S15. Multiple linear regression of sleep-related problem changes and depressive symptom changes (n=1405)**

| **Variable** | **Classification** | **Model 1** | | **Model 2** | | **Model 3** | |
| --- | --- | --- | --- | --- | --- | --- | --- |
|  |  | ***β* (95% *CI*)** | ***P*** | ***β* (95% *CI*)** | ***P*** | ***β* (95% *CI*)** | ***P*** |
| **∆PSQI** |  | 0.336 (0.269-0.404) | <0.001 | 0.346 (0.279-0.414) | <0.001 | 0.348 (0.280-0.416） | <0.001 |
| **Baseline PSQI-5** |  | 0.306 (0.232-0.381) | <0.001 | 0.306 (0.231-0.382) | **<0.001** | 0.305 (0.251-0.381） | <0.001 |
| **Baseline CDI-S** |  | -0.547 (-0.590--0.504) | <0.001 | -0.559 (-0.603--0.516) | **<0.001** | -0.560 (-0.601--0.516) | <0.001 |
| **Sex** (Male=0) | Female |  |  | -0.008 (-0.256-0.239) | 0.949 | -0.008 (-0.259-0.244) | 0.953 |
| **School** (School A=0) | School B |  |  | 0.253 (-0.083-0.589) | 0.139 | 0.252 (-0.084-0.588) | 0.141 |
|  | School C |  |  | 0.020 (-0.435-0.475) | 0.931 | 0.040 (-0.416-0.495) | 0.865 |
| **Grade** (Grade 1=0) | Grade 2 |  |  | -0.331 (-0.664-0.001) | 0.051 | -0.291 (-0.629-0.046) | 0.091 |
|  | Grade 3 |  |  | -0.645 (-0.970--0.319) | <0.001 | -0.554 (-0.909--0.200) | 0.002 |
| **Residence** (Urban=0) | Rural |  |  | -0.037 (-0.669-0.596) | 0.909 | -0.044 (-0.677-0.590) | 0.892 |
| **Parental divorce** (No=0) | Yes |  |  | 0.028 (-0.304-0.359) | 0.870 | 0.029 (-0.303-0.361) | 0.863 |
| **Father/mother deceased** (No=0) | Yes |  |  | -0.344 (-1.833-1.146) | 0.651 | -0.367 (-1.857-1.124) | 0.630 |
| **Family structure**  (Two-parent family=0) | Single mother family |  |  | 0.343 (-0.107-0.792) | 0.135 | 0.345 (-0.105-0.795) | 0.132 |
|  | Single father family |  |  | 0.592 (-0.203-1.387) | 0.144 | 0.601 (-0.196-1.397) | 0.139 |
|  | Both parents absent |  |  | 0.998 (0.107-1.889) | 0.028 | 0.961 (0.067-1.854) | **0.035** |
| **Only child** (No=0) | Yes |  |  | -0.190 (-0.448-0.068) | 0.148 | -0.179 (-0.437-0.079) | 0.174 |
| **Father's education level**  (Primary school or below=0) | Junior high school |  |  | 0.866 (-0.368-2.099) | 0.169 | 0.901 (-0.334-2.135) | 0.152 |
|  | High school or technical secondary school |  |  | 0.809 (-0.431-2.048) | 0.201 | 0.843 (-0.399-2.085) | 0.183 |
|  | Bachelor or associate degree |  |  | 0.846 (-0.403-2.096) | 0.184 | 0.881 (-0.371-2.132) | 0.168 |
|  | Master's degree or above |  |  | 0.816 (-0.591-2.224) | 0.255 | 0.827 (-0.581-2.235) | 0.249 |
| **Mother's education level**  (Primary school or below=0) | Junior high school |  |  | -0.727 (-2.164-0.710) | 0.321 | -0.678 (-2.116-0.761) | 0.355 |
|  | High school or technical secondary school |  |  | -0.808 (-2.247-0.630) | 0.271 | -0.778 (-2.218-0.661) | 0.289 |
|  | Bachelor or associate degree |  |  | -1.016 (-2.461-0.430) | 0.168 | -0.978 (-2.425-0.469) | 0.185 |
|  | Master's degree or above |  |  | -1.066 (-2.689-0.557) | 0.198 | -1.029 (-2.652-0.595) | 0.214 |
| **Monthly household income per capita (yuan)** (<2000=0) | 2000-4000 |  |  | -0.381 (-1.146-0.384) | 0.329 | -0.424 (-1.192-0.344) | 0.279 |
|  | 4000-6000 |  |  | -0.360 (-1.141-0.422) | 0.367 | -0.386 (-1.170-0.397) | 0.333 |
|  | 6000-8000 |  |  | -0.248 (-1.038-0.541) | 0.537 | -0.265 (-1.056-0.527) | 0.512 |
|  | 8000-10000 |  |  | -0.547 (-1.338-0.244) | 0.175 | -0.558 (-1.352-0.235) | 0.168 |
|  | >10000 |  |  | -0.092 (-0.868-0.684) | 0.816 | 0.111 (-0.889-0.668) | 0.780 |
| **Chronic disease** (No=0) | Yes |  |  |  |  | -0.733 (-1.518-0.052) | 0.067 |
| **Weight（kg）** |  |  |  |  |  | -0.013 (-0.034-0.008) | 0.223 |
| **Prefer sleeping with light on** (No=0) | Yes |  |  |  |  | -0.004 (-0.368-0.359) | 0.981 |
| **Eating after 9 PM** (Never=0) | Occasionally |  |  |  |  | 0.003 (-0.314-0.320) | 0.986 |
|  | Every day |  |  |  |  | 0.113 (-0.674-0.899) | 0.778 |

*Note*: Model 1 adjusted for ∆PSQI, baseline PSQI-5, and corresponding baseline symptom levels; Model 2 further adjusted for demographic and family socioeconomic factors based on Model 1; Model 3 further adjusted for health and behavioral factors based on Model 2.
