# Supplementary material for: Study on the association between sleep quality and anxiety–depression symptoms in school-aged children
Source: Front Public Health. 2026 Oct 20;14:1927475. doi: 10.3389/fpubh.2026.1927475 (PMC13727519; doi:10.3389/fpubh.2026.1927475)
Supplement: Supplementary file 8 [file Table_2.DOCX]

**Table S2. Multiple linear regression of sleep-related problem changes and follow-up anxiety symptoms (n=1529)**

| **Variable** | **Classification** | **Model 1** | | **Model 2** | | **Model 3** | |
| --- | --- | --- | --- | --- | --- | --- | --- |
|  |  | ***β* (95% *CI*)** | ***P*** | ***β* (95% *CI*)** | ***P*** | ***β* (95% *CI*)** | ***P*** |
| **∆PSQI** |  | 0.893 (0.806-0.981) | <0.001 | 0.896 (0.807-0.984) | <0.001 | 0.896 (0.808-0.985) | <0.001 |
| **Baseline PSQI-5** |  | 0.775 (0.671-0.878) | <0.001 | 0.766 (0.661-0.871) | <0.001 | 0.765 (0.660-0.871) | <0.001 |
| **Baseline GAD-7** |  | 0.348 (0.303-0.393) | <0.001 | 0.344 (0.298-0.389) | <0.001 | 0.344 (0.298-0.390) | <0.001 |
| **Sex** (Male=0) | Female |  |  | -0.165 (-0.488-0.159) | 0.318 | -0.171 (-0.501-0.159) | 0.309 |
| **School** (School A=0) | School B |  |  | 0.269 (-0.174-0.711) | 0.235 | 0.269 (-0.175-0.712) | 0.235 |
|  | School C |  |  | 0.277 (-0.301-0.856) | 0.347 | 0.300 (-0.282-0.881) | 0.312 |
| **Grade** (Grade 1=0) | Grade 2 |  |  | -0.149 (-0.584-0.285) | 0.500 | -0.111 (-0.553-0.331) | 0.622 |
|  | Grade 3 |  |  | -0.228 (-0.653-0.197) | 0.293 | -0.154 (-0.620-0.312) | 0.516 |
| **Residence** (Urban=0) | Rural |  |  | 0.112 (-0.709-0.933) | 0.790 | 0.141 (-0.684-0.965) | 0.738 |
| **Parental divorce** (No=0) | Yes |  |  | 0.326 (-0.125-0.776) | 0.156 | 0.345 (-0.107-0.796) | 0.134 |
| **Father/mother deceased** (No=0) | Yes |  |  | 0.104 (-1.690-1.898) | 0.909 | 0.097 (-1.648-1.843) | 0.913 |
| **Family structure**  (Two-parent family=0) | Single mother family |  |  | 0.420 (-0.185-1.025) | 0.174 | 0.402 (-0.205-1.009) | 0.194 |
|  | Single father family |  |  | 0.467 (-0.553-1.488) | 0.369 | 0.454 (-0.574-1.483) | 0.386 |
|  | Both parents absent |  |  | 0.958 (-0.225-2.141) | 0.112 | 0.886 (-0.296-2.068) | 0.142 |
| **Only child** (No=0) | Yes |  |  | -0.503 (-0.858--0.147) | 0.006 | -0.506 (-0.863--0.149) | 0.005 |
| **Father's education level**  (Primary school or below=0) | Junior high school |  |  | 0.207 (-1.122-1.535) | 0.760 | 0.225 (-1.089-1.539) | 0.737 |
|  | High school or technical secondary school |  |  | -0.255 (-1.569-1.059) | 0.703 | -0.249 (-1.553-1.054) | 0.707 |
|  | Bachelor or associate degree |  |  | -0.245 (-1.574-1.084) | 0.717 | -0.255 (-1.577-1.066) | 0.704 |
|  | Master's degree or above |  |  | -0.332 (-1.882-1.218) | 0.674 | -0.361 (-1.892-1.171) | 0.644 |
| **Mother's education level**  (Primary school or below=0) | Junior high school |  |  | -1.046 (-2.812-0.720) | 0.245 | -1.037 (-2.798-0.723) | 0.248 |
|  | High school or technical secondary school |  |  | -0.672 (-2.449-1.105) | 0.458 | -0.671 (-2.442-1.100) | 0.457 |
|  | Bachelor or associate degree |  |  | -0.782 (-2.548-0.984) | 0.385 | -0.759 (-2.522-1.005) | 0.399 |
|  | Master's degree or above |  |  | -0.695 (-2.635-1.246) | 0.483 | -0.691 (-2.628-1.246) | 0.484 |
| **Monthly household income per capita (yuan)** (<2000=0) | 2000-4000 |  |  | 0.343 (-0.651-1.338) | 0.498 | 0.362 (-0.637-1.362) | 0.477 |
|  | 4000-6000 |  |  | 0.138 (-0.887-1.163) | 0.791 | 0.158 (-0.871-1.186) | 0.763 |
|  | 6000-8000 |  |  | 0.254 (-0.771-1.280) | 0.626 | 0.284 (-0.744-1.312) | 0.589 |
|  | 8000-10000 |  |  | -0.151 (-1.181-0.879) | 0.774 | -0.119 (-1.153-0.914) | 0.821 |
|  | >10000 |  |  | 0.160 (-0.858-1.178) | 0.758 | 0.181 (-0.839-1.201) | 0.727 |
| **Chronic disease** (No=0) | Yes |  |  |  |  | -0.351 (-1.420-0.719) | 0.520 |
| **Weight (kg)** |  |  |  |  |  | -0.010 (-0.037-0.018) | 0.485 |
| **Prefer sleeping with light on** (No=0) | Yes |  |  |  |  | -0.335 (-0.828-0.158) | 0.183 |
| **Eating after 9 PM** (Never=0) | Occasionally |  |  |  |  | -0.198 (-0.632-0.237) | 0.373 |
|  | Every day |  |  |  |  | -0.066 (-1.163-1.032) | 0.907 |

*Note*: Model 1 adjusted for ∆PSQI, baseline PSQI-5, and corresponding baseline symptom levels; Model 2 further adjusted for demographic and family socioeconomic factors based on Model 1; Model 3 further adjusted for health and behavioral factors based on Model 2. Missing covariates were handled by multiple imputation (full conditional specification method, m=20), and the imputation results were combined for inference.
