# Supplementary material for: Study on the association between sleep quality and anxiety–depression symptoms in school-aged children
Source: Front Public Health. 2026 Oct 20;14:1927475. doi: 10.3389/fpubh.2026.1927475 (PMC13727519; doi:10.3389/fpubh.2026.1927475)
Supplement: Supplementary file 9 [file Table_3.DOCX]

**Table S3. Multiple linear regression of sleep-related problem changes and follow-up depressive symptoms (n=1529)**

| **Variable** | **Classification** | **Model 1** | | **Model 2** | | **Model 3** | |
| --- | --- | --- | --- | --- | --- | --- | --- |
|  |  | ***β* (95% *CI*)** | ***P*** | ***β* (95% *CI*)** | ***P*** | ***β* (95% *CI*)** | ***P*** |
| **∆PSQI** |  | 0.349 (0.284-0.414) | <0.001 | 0.346 (0.279-0.414) | <0.001 | 0.348 (0.280-0.415) | <0.001 |
| **Baseline PSQI-5** |  | 0.323 (0.251-0.394) | <0.001 | 0.306 (0.231-0.382) | <0.001 | 0.305 (0.230-0.381) | <0.001 |
| **Baseline CDI-S** |  | 0.451 (0.410-0.492) | <0.001 | 0.441 (0.397-0.484) | <0.001 | 0.440 (0.397-0.484) | <0.001 |
| **Sex** (Male=0) | Female |  |  | 0.008 (-0.239-0.255) | 0.949 | 0.008 (-0.244-0.259) | 0.953 |
| **School** (School A=0) | School B |  |  | 0.253 (-0.082-0.588) | 0.139 | 0.252 (-0.083-0.588) | 0.141 |
|  | School C |  |  | 0.020 (-0.435-0.475) | 0.931 | 0.040 (-0.416-0.495) | 0.865 |
| **Grade** (Grade 1=0) | Grade 2 |  |  | -0.331 (-0.664-0.001) | 0.051 | -0.291 (-0.628-0.046) | 0.090 |
|  | Grade 3 |  |  | -0.645 (-0.970--0.319) | <0.001 | -0.554 (-0.909--0.200) | 0.002 |
| **Residence** (Urban=0) | Rural |  |  | -0.037 (-0.669-0.595) | 0.909 | -0.044 (-0.677-0.589) | 0.892 |
| **Parental divorce** (No=0) | Yes |  |  | 0.028 (-0.304-0.359) | 0.870 | 0.029 (-0.303-0.361) | 0.863 |
| **Father/mother deceased** (No=0) | Yes |  |  | -0.344 (-1.832-1.145) | 0.651 | -0.367 (-1.856-1.123) | 0.630 |
| **Family structure**  (Two-parent family=0) | Single mother family |  |  | 0.343 (-0.106-0.791) | 0.135 | 0.345 (-0.104-0.794) | 0.132 |
|  | Single father family |  |  | 0.592 (-0.202-1.386) | 0.144 | 0.601 (-0.195-1.397) | 0.139 |
|  | Both parents absent |  |  | 0.998 (0.108-1.888) | 0.028 | 0.961 (0.068-1.853) | 0.035 |
| **Only child** (No=0) | Yes |  |  | -0.190 (-0.448-0.067) | 0.148 | -0.179 (-0.437-0.079) | 0.174 |
| **Father's education level**  (Primary school or below=0) | Junior high school |  |  | 0.866 (-0.367-2.098) | 0.169 | 0.901 (-0.332-2.134) | 0.152 |
|  | High school or technical secondary school |  |  | 0.809 (-0.430-2.047) | 0.201 | 0.843 (-0.398-2.084) | 0.183 |
|  | Bachelor or associate degree |  |  | 0.846 (-0.402-2.095) | 0.184 | 0.881 (-0.370-2.131) | 0.167 |
|  | Master's degree or above |  |  | 0.816 (-0.590-2.223) | 0.255 | 0.827 (-0.580-2.234) | 0.249 |
| **Mother's education level**  (Primary school or below=0) | Junior high school |  |  | -0.727 (-2.162-0.709) | 0.321 | -0.678 (-2.115-0.759) | 0.355 |
|  | High school or technical secondary school |  |  | -0.808 (-2.246-0.629) | 0.270 | -0.778 (-2.216-0.660) | 0.289 |
|  | Bachelor or associate degree |  |  | -1.016 (-2.460-0.428) | 0.168 | -0.978 (-2.424-0.467) | 0.185 |
|  | Master's degree or above |  |  | -1.066 (-2.688-0.555) | 0.197 | -1.029 (-2.651-0.594) | 0.214 |
| **Monthly household income per capita (yuan)** (<2000=0) | 2000-4000 |  |  | -0.381 (-1.145-0.383) | 0.329 | -0.424 (-1.192-0.343) | 0.278 |
|  | 4000-6000 |  |  | -0.360 (-1.140-0.421) | 0.367 | -0.386 (-1.169-0.396) | 0.333 |
|  | 6000-8000 |  |  | -0.248 (-1.037-0.540) | 0.537 | -0.265 (-1.055-0.526) | 0.512 |
|  | 8000-10000 |  |  | -0.547 (-1.338-0.244) | 0.175 | -0.558 (-1.351-0.234) | 0.167 |
|  | >10000 |  |  | -0.092 (-0.868-0.683) | 0.816 | -0.111 (-0.888-0.667) | 0.780 |
| **Chronic disease** (No=0) | Yes |  |  |  |  | -0.733 (-1.517-0.052) | 0.067 |
| **Weight (kg)** |  |  |  |  |  | -0.013 (-0.034-0.008) | 0.222 |
| **Prefer sleeping with light on** (No=0) | Yes |  |  |  |  | -0.004 (-0.368-0.359) | 0.981 |
| **Eating after 9 PM** (Never=0) | Occasionally |  |  |  |  | 0.003 (-0.314-0.319) | 0.986 |
|  | Every day |  |  |  |  | 0.113 (-0.673-0.899) | 0.778 |

*Note*: Model 1 adjusted for ∆PSQI, baseline PSQI-5, and corresponding baseline symptom levels; Model 2 further adjusted for demographic and family socioeconomic factors based on Model 1; Model 3 further adjusted for health and behavioral factors based on Model 2. Missing covariates were handled by multiple imputation (full conditional specification method, m=20), and the imputation results were combined for inference.
