# Supplementary material for: Study on the association between sleep quality and anxiety–depression symptoms in school-aged children
Source: Front Public Health. 2026 Oct 20;14:1927475. doi: 10.3389/fpubh.2026.1927475 (PMC13727519; doi:10.3389/fpubh.2026.1927475)
Supplement: Supplementary file 11 [file Table_5.DOCX]

**Table S5. Multiple linear regression of sleep-related problem changes and depressive symptom changes (n=1529)**

| **Variable** | **Classification** | **Model 1** | | **Model 2** | | **Model 3** | |
| --- | --- | --- | --- | --- | --- | --- | --- |
|  |  | ***β* (95% *CI*)** | ***P*** | ***β* (95% *CI*)** | ***P*** | ***β* (95% *CI*)** | ***P*** |
| **∆PSQI** |  | 0.349 (0.284-0.414) | <0.001 | 0.358 (0.293-0.423) | <0.001 | 0.359 (0.294-0.424) | <0.001 |
| **Baseline PSQI-5** |  | 0.323 (0.251-0.394) | <0.001 | 0.325 (0.253-0.398) | <0.001 | 0.324 (0.251-0.397) | <0.001 |
| **Baseline CDI-S** |  | -0.549 (-0.590--0.508) | <0.001 | -0.560 (-0.601--0.518) | <0.001 | -0.559 (-0.601--0.518) | <0.001 |
| **Sex** (Male=0) | Female |  |  | 0.092 (-0.146-0.331) | 0.448 | 0.086 (-0.156-0.328) | 0.487 |
| **School** (School A=0) | School B |  |  | 0.242 (-0.084-0.568) | 0.145 | 0.241 (-0.085-0.567) | 0.148 |
|  | School C |  |  | 0.079 (-0.346-0.503) | 0.717 | 0.101 (-0.324-0.526) | 0.642 |
| **Grade** (Grade 1=0) | Grade 2 |  |  | -0.297 (-0.616-0.022) | 0.068 | -0.245 (-0.570-0.079) | 0.138 |
|  | Grade 3 |  |  | -0.593 (-0.905--0.281) | <0.001 | -0.478 (-0.820--0.136) | 0.006 |
| **Residence** (Urban=0) | Rural |  |  | -0.040 (-0.640-0.560) | 0.896 | -0.046 (-0.646-0.554) | 0.880 |
| **Parental divorce** (No=0) | Yes |  |  | 0.018 (-0.315-0.351) | 0.917 | 0.018 (-0.315-0.351) | 0.915 |
| **Father/mother deceased** (No=0) | Yes |  |  | -0.237 (-1.527-1.054) | 0.717 | -0.284 (-1.564-0.997) | 0.663 |
| **Family structure**  (Two-parent family=0) | Single mother family |  |  | 0.324 (-0.138-0.786) | 0.169 | 0.328 (-0.134-0.790) | 0.165 |
|  | Single father family |  |  | 0.541 (-0.257-1.339) | 0.183 | 0.550 (-0.251-1.350) | 0.178 |
|  | Both parents absent |  |  | 0.907 (0.030-1.784) | 0.043 | 0.862 (-0.019-1.743) | 0.055 |
| **Only child** (No=0) | Yes |  |  | -0.167 (-0.427-0.092) | 0.207 | -0.156 (-0.415-0.104) | 0.240 |
| **Father's education level**  (Primary school or below=0) | Junior high school |  |  | 0.289 (-0.810-1.389) | 0.603 | 0.330 (-0.763-1.424) | 0.551 |
|  | High school or technical secondary school |  |  | 0.212 (-0.889-1.313) | 0.704 | 0.249 (-0.848-1.345) | 0.654 |
|  | Bachelor or associate degree |  |  | 0.256 (-0.849-1.360) | 0.648 | 0.293 (-0.808-1.395) | 0.599 |
|  | Master's degree or above |  |  | 0.255 (-0.993-1.504) | 0.687 | 0.275 (-0.973-1.524) | 0.664 |
| **Mother's education level**  (Primary school or below=0) | Junior high school |  |  | -0.241 (-1.582-1.099) | 0.724 | -0.198 (-1.538-1.142) | 0.772 |
|  | High school or technical secondary school |  |  | -0.303 (-1.664-1.057) | 0.661 | -0.280 (-1.638-1.078) | 0.685 |
|  | Bachelor or associate degree |  |  | -0.503 (-1.862-0.856) | 0.467 | -0.472 (-1.832-0.888) | 0.496 |
|  | Master's degree or above |  |  | -0.490 (-2.011-1.030) | 0.527 | -0.467 (-1.988-1.054) | 0.546 |
| **Monthly household income per capita (yuan)** (<2000=0) | 2000-4000 |  |  | -0.277 (-1.006-0.451) | 0.456 | -0.316 (-1.052-0.419) | 0.399 |
|  | 4000-6000 |  |  | -0.219 (-0.956-0.517) | 0.560 | -0.239 (-0.981-0.503) | 0.527 |
|  | 6000-8000 |  |  | -0.125 (-0.866-0.617) | 0.742 | -0.131 (-0.877-0.615) | 0.731 |
|  | 8000-10000 |  |  | -0.417 (-1.161-0.326) | 0.271 | -0.420 (-1.168-0.328) | 0.271 |
|  | >10000 |  |  | 0.017 (-0.706-0.739) | 0.964 | 0.006 (-0.721-0.733) | 0.987 |
| **Chronic disease** (No=0) | Yes |  |  |  |  | -0.757 (-1.545-0.030) | 0.059 |
| **Weight (kg)** |  |  |  |  |  | -0.016 (-0.036-0.004) | 0.122 |
| **Prefer sleeping with light on** (No=0) | Yes |  |  |  |  | -0.014 (-0.381-0.352) | 0.938 |
| **Eating after 9 PM** (Never=0) | Occasionally |  |  |  |  | -0.005 (-0.329-0.318) | 0.975 |
|  | Every day |  |  |  |  | 0.057 (-0.681-0.796) | 0.880 |

*Note*: Model 1 adjusted for ∆PSQI, baseline PSQI-5, and corresponding baseline symptom levels; Model 2 further adjusted for demographic and family socioeconomic factors based on Model 1; Model 3 further adjusted for health and behavioral factors based on Model 2. Missing covariates were handled by multiple imputation (full conditional specification method, m=20), and the imputation results were combined for inference.
