# Supplementary material for: Study on the association between sleep quality and anxiety–depression symptoms in school-aged children
Source: Front Public Health. 2026 Oct 20;14:1927475. doi: 10.3389/fpubh.2026.1927475 (PMC13727519; doi:10.3389/fpubh.2026.1927475)
Supplement: Supplementary file 12 [file Table_6.DOCX]

**Table S6. Baseline characteristics of participants with and without missing covariate data**

| **Variable** | **Complete data group (n=1 405)** | | **Missing data group (n=124)** | | ***t* / *χ²*** | ***P*** |
| --- | --- | --- | --- | --- | --- | --- |
|  | **Mean (SD)** | **n (%)** | **Mean (SD)** | **n (%)** |  |  |
| **Baseline PSQI-5 total scores** | 4.79 (2.12) |  | 4.65 (2.11) |  | 0.707 | 0.480 |
| **Baseline GAD-7 total scores** | 4.48(4.17) |  | 4.53 (4.57) |  | -0.121 | 0.904 |
| **Baseline CDI-S total scores** | 4.56 (3.18) |  | 4.98 (3.61) |  | -1.381 | 0.167 |
| **Sociodemographic characteristics** |  |  |  |  |  |  |
| **Age (years)** | 7.99 (1.03) |  | 7.92 (1.12) |  | 0.547 | 0.585 |
| **Sex** |  |  |  |  | 0.071 | 0.790 |
| Male |  | 720 (51.2) |  | 62 (50.0) |  |  |
| Female |  | 685 (48.8) |  | 62 (50.0) |  |  |
| **Weight (kg)** |  |  |  |  | 0.033 | 0.974 |
| **Grade** |  |  |  |  | 3.294 | 0.193 |
| Grade 1 |  | 386 (27.5) |  | 43 (34.7) |  |  |
| Grade 2 |  | 452 (32.2) |  | 33 (26.6) |  |  |
| Grade 3 |  | 567 (40.4) |  | 48 (38.7) |  |  |
| Grade 4 |  |  |  |  |  |  |
| **School** |  |  |  |  | 93.546 | <0.001 |
| School A |  | 320 (22.8) |  | 28 (22.6) |  |  |
| School B |  | 804 (57.2) |  | 26 (21.0) |  |  |
| School C |  | 281 (20.0) |  | 70 (56.5) |  |  |
| **Residence** |  |  |  |  | 0.099 | 0.754 |
| Urban |  | 1339 (95.3) |  | 2 (100.0) |  |  |
| Rural |  | 66 (4.7) |  | 0 (0.0) |  |  |
| **Father's education level** |  |  |  |  | 10.593 | **0.032** |
| Primary school or below |  | 20 (1.4) |  | 0 (0.0) |  |  |
| Junior high school |  | 219 (15.6) |  | 1 (50.0) |  |  |
| High school or technical secondary school |  | 355 (25.3) |  | 0 (0.0) |  |  |
| Bachelor or associate degree |  | 737 (52.5) |  | 0 (0.0) |  |  |
| Master's degree or above |  | 74 (5.3) |  | 1 (50.0) |  |  |
| **Mother's education level** |  |  |  |  | 2.191 | 0.701 |
| Primary school or below |  | 14 (1.0) |  | 0 (0.0) |  |  |
| Junior high school |  | 215 (15.3) |  | 1 (50.0) |  |  |
| High school or technical secondary school |  | 365 (26.0) |  | 0 (0.0) |  |  |
| Bachelor or associate degree |  | 755 (53.7) |  | 1 (50.0) |  |  |
| Master's degree or above |  | 56 (4.0) |  | 0 (0.0) |  |  |
| **Monthly household income per capita (yuan)** |  |  |  |  | 16.457 | **0.006** |
| <2000 |  | 45 (3.2) |  | 1 (50.0) |  |  |
| 2000-4000 |  | 283 (20.1) |  | 0 (0.0) |  |  |
| 4000-6000 |  | 232 (16.6) |  | 0 (0.0) |  |  |
| 6000-8000 |  | 223 (15.9) |  | 1 (50.0) |  |  |
| 8000-10000 |  | 240 (17.1) |  | 0 (0.0) |  |  |
| >10000 |  | 382 (27.2) |  | 0 (0.0) |  |  |
| **Family structure** |  |  |  |  | 22.869 | <0.001 |
| Two-parent family |  | 1217 (86.6) |  | 1 (50.0) |  |  |
| Single mother family |  | 124 (8.8) |  | 0 (0.0) |  |  |
| Single father family |  | 36 (2.6) |  | 0 (0.0) |  |  |
| Both parents absent |  | 28 (2.0) |  | 1 (50.0) |  |  |
| **Only child** |  |  |  |  | - | - |
| Yes |  | 865 (61.6) |  | 0 (0.0) |  |  |
| No |  | 540 (38.4) |  | 0 (0.0) |  |  |
| **Father/mother deceased** |  |  |  |  | 0.014 | 0.905 |
| Yes |  | 10 (0.7) |  | 0 (0.0) |  |  |
| No |  | 1395 (99.3) |  | 2 (100.0) |  |  |
| **Parental divorce** |  |  |  |  | 0.815 | 0.367 |
| Yes |  | 998 (71.0) |  | 2 (100.0) |  |  |
| No |  | 407 (29.0) |  | 0 (0.0) |  |  |
| **Chronic disease** |  |  |  |  | 3.161 | 0.075 |
| Yes |  | 35 (2.5) |  | 0 (0.0) |  |  |
| No |  | 1370 (97.5) |  | 124 (100.0) |  |  |
| **Prefer sleeping with light on** |  |  |  |  | 0.301 | 0.583 |
| Yes |  | 184 (13.1) |  | 0 (0.0) |  |  |
| No |  | 1221 (86.9) |  | 2 (100.0) |  |  |
| **Eating after 9 PM** |  |  |  |  | 1.314 | 0.518 |
| Never |  | 262 (18.6) |  | 1 (50.0) |  |  |
| Occasionally |  | 1103 (78.5) |  | 1 (50.0) |  |  |
| Every day |  | 40 (2.8) |  | 0 (0.0) |  |  |

*Note*: Data are presented as mean (SD) or n (%). *P* values: *t*-test for continuous variables, *χ²* test for categorical variables (complete vs. missing groups). Percentages were based on non-missing cases.
