# Supplementary material for: Study on the association between sleep quality and anxiety–depression symptoms in school-aged children
Source: Front Public Health. 2026 Oct 20;14:1927475. doi: 10.3389/fpubh.2026.1927475 (PMC13727519; doi:10.3389/fpubh.2026.1927475)
Supplement: Supplementary file 13 [file Table_7.DOCX]

**Table S7. Longitudinal association between baseline sleep-related problems and follow-up positive anxiety symptoms (n=1405)**

| **Variable** | **Classification** | **Model 1** | | **Model 2** | | **Model 3** | | **Model 4** | |
| --- | --- | --- | --- | --- | --- | --- | --- | --- | --- |
|  |  | ***OR* (95% *CI*)** | ***P*** | ***OR* (95% *CI*)** | ***P*** | ***OR* (95% *CI*)** | ***P*** | ***OR* (95% *CI*)** | ***P*** |
| **Baseline PSQI-5** |  | 1.379 (1.302-1.462) | <0.001 | 1.362 (1.283-1.444) | <0.001 | 1.361 (1.283-1.444) | <0.001 | 1.215 (1.140-1.295) | <0.001 |
| **Sex** (Male=0) | Female |  |  | 0.730 (0.576-0.925) | 0.009 | 0.719 (0.564-0.916) | 0.008 | 0.708 (0.550-0.912) | 0.008 |
| **School** (School A=0) | School B |  |  | 0.921 (0.672-1.264) | 0.611 | 0.919 (0.670-1.262) | 0.602 | 0.896 (0.643-1.249) | 0.516 |
|  | School C |  |  | 0.672 (0.434-1.040) | 0.075 | 0.677 (0.437-1.049) | 0.081 | 0.666 (0.422-1.050) | 0.080 |
| **Grade** (Grade 1=0) | Grade 2 |  |  | 0.821 (0.597-1.128) | 0.223 | 0.826 (0.598-1.140) | 0.245 | 0.905 (0.647-1.267) | 0.561 |
|  | Grade 3 |  |  | 0.788 (0.578-1.076) | 0.134 | 0.826 (0.588-1.160) | 0.270 | 0.935 (0.656-1.333) | 0.709 |
| **Residence** (Urban=0) | Rural |  |  | 1.192 (0.656-2.166) | 0.565 | 1.193 (0.656-2.173) | 0.563 | 1.246 (0.667-2.325) | 0.490 |
| **Parental divorce** (No=0) | Yes |  |  | 1.089 (0.793-1.495) | 0.599 | 1.106 (0.805-1.519) | 0.535 | 1.120 (0.803-1.562) | 0.504 |
| **Father/mother deceased** (No=0) | Yes |  |  | 1.070 (0.275-4.163) | 0.922 | 1.074 (0.277-4.162) | 0.918 | 1.416 (0.343-5.850) | 0.631 |
| **Family structure**  (Two-parent family=0) | Single mother family |  |  | 1.332 (0.880-2.016) | 0.176 | 1.333 (0.880-2.020) | 0.175 | 1.085 (0.700-1.683) | 0.716 |
|  | Single father family |  |  | 1.526 (0.731-3.188) | 0.260 | 1.501 (0.718-3.138) | 0.280 | 1.403 (0.648-3.038) | 0.390 |
|  | Both parents absent |  |  | 1.516 (0.658-3.492) | 0.329 | 1.489 (0.644-3.439) | 0.352 | 1.452 (0.624-3.378) | 0.387 |
| **Only child** (No=0) | Yes |  |  | 0.876 (0.683-1.122) | 0.295 | 0.869 (0.678-1.115) | 0.270 | 0.866 (0.667-1.124) | 0.279 |
| **Father's education level**  (Primary school or below=0) | Junior high school |  |  | 0.611 (0.195-1.916) | 0.399 | 0.616 (0.197-1.926) | 0.405 | 0.693 (0.217-2.213) | 0.536 |
|  | High school or technical secondary school |  |  | 0.451 (0.143-1.422) | 0.174 | 0.456 (0.145-1.438) | 0.180 | 0.474 (0.148-1.522) | 0.210 |
|  | Bachelor or associate degree |  |  | 0.372 (0.117-1.186) | 0.095 | 0.375 (0.118-1.194) | 0.097 | 0.389 (0.120-1.264) | 0.116 |
|  | Master's degree or above |  |  | 0.389 (0.105-1.450) | 0.160 | 0.377 (0.101-1.403) | 0.146 | 0.398 (0.104-1.522) | 0.178 |
| **Mother's education level**  (Primary school or below=0) | Junior high school |  |  | 0.962 (0.251-3.683) | 0.954 | 0.993 (0.260-3.794) | 0.992 | 0.771 (0.197-3.022) | 0.709 |
|  | High school or technical secondary school |  |  | 1.342 (0.351-5.132) | 0.667 | 1.376 (0.361-5.242) | 0.640 | 1.095 (0.280-4.273) | 0.897 |
|  | Bachelor or associate degree |  |  | 1.199 (0.311-4.623) | 0.792 | 1.239 (0.322-4.766) | 0.755 | 0.967 (0.245-3.811) | 0.962 |
|  | Master's degree or above |  |  | 1.493 (0.326-6.839) | 0.605 | 1.549 (0.3391-7.070) | 0.572 | 1.158 (0.245-5.479) | 0.853 |
| **Monthly household income per capita (yuan)** (<2000=0) | 2000-4000 |  |  | 1.085 (0.524-2.243) | 0.827 | 1.130 (0.544-2.347) | 0.743 | 1.216 (0.565-2.621) | 0.617 |
|  | 4000-6000 |  |  | 1.033 (0.491-2.174) | 0.932 | 1.071 (0.507-2.263) | 0.857 | 1.076 (0.491-2.360) | 0.855 |
|  | 6000-8000 |  |  | 1.168 (0.551-2.474) | 0.686 | 1.218 (0.573-2.590) | 0.608 | 1.394 (0.631-3.077) | 0.412 |
|  | 8000-10000 |  |  | 0.879 (0.412-1.875) | 0.738 | 0.912 (0.426-1.953) | 0.813 | 1.003 (0.451-2.231) | 0.995 |
|  | >10000 |  |  | 1.039 (0.495-2.178) | 0.920 | 1.077 (0.512-2.266) | 0.845 | 1.113 (0.509-2.432) | 0.789 |
| **Chronic disease** (No=0) | Yes |  |  |  |  | 1.071 (0.500-2.294) | 0.860 | 1.030 (0.462-2.296) | 0.943 |
| **Weight（kg）** |  |  |  |  |  | 0.992 (0.973-1.013) | 0.458 | 0.989 (0.968-1.010) | 0.301 |
| **Prefer sleeping with light on** (No=0) | Yes |  |  |  |  | 1.032 (0.729-1.462) | 0.858 | 1.031 (0.716-1.484) | 0.868 |
| **Eating after 9 PM** (Never=0) | Occasionally |  |  |  |  | 0.834 (0.616-1.127) | 0.238 | 0.840 (0.615-1.148) | 0.274 |
|  | Every day |  |  |  |  | 1.246 (0.600-2.587) | 0.556 | 1.216 (0.564-2.621) | 0.618 |
| **Baseline GAD-7** |  |  |  |  |  |  |  | 3.873 (2.989-5.019) | <0.001 |

*Note*: Model 1 is the unadjusted model; Model 2 adjusts for demographic and family socio-economic factors; Model 3 further adjusts for health and behavioral factors based on Model 2; Model 4 further incorporates the corresponding baseline symptom levels based on Model 3.
