# Supplementary material for: Study on the association between sleep quality and anxiety–depression symptoms in school-aged children
Source: Front Public Health. 2026 Oct 20;14:1927475. doi: 10.3389/fpubh.2026.1927475 (PMC13727519; doi:10.3389/fpubh.2026.1927475)
Supplement: Supplementary file 14 [file Table_8.DOCX]

**Table S8. Longitudinal association between baseline sleep-related problems and follow-up positive depressive symptoms (n=1405)**

| **Variable** | **Classification** | **Model 1** | | **Model 2** | | **Model 3** | | **Model 4** | |
| --- | --- | --- | --- | --- | --- | --- | --- | --- | --- |
|  |  | ***OR* (95% *CI*)** | ***P*** | ***OR* (95% *CI*)** | ***P*** | ***OR* (95% *CI*)** | ***P*** | ***OR* (95% *CI*)** | ***P*** |
| **Baseline PSQI-5** |  | 1.327 (1.245-1.414) | <0.001 | 1.316 (1.232-1.407) | <0.001 | 1.318 (1.233-1.408) | <0.001 | 1.170 (1.088-1.259) | <0.001 |
| **Sex** (Male=0) | Female |  |  | 0.766 (0.572-1.027) | 0.075 | 0.774 (0.575-1.043) | 0.092 | 0.798 (0.582-1.095) | 0.162 |
| **School** (School A=0) | School B |  |  | 1.397 (0.933-2.092) | 0.105 | 1.404 (0.938-2.102) | 0.100 | 1.386 (0.902-2.130) | 0.136 |
|  | School C |  |  | 0.813 (0.467-1.414) | 0.463 | 0.821 (0.472-1.429) | 0.486 | 0.860 (0.478-1.547) | 0.615 |
| **Grade** (Grade 1=0) | Grade 2 |  |  | 0.705 (0.484-1.026) | 0.068 | 0.707 (0.483-1.036) | 0.075 | 0.716 (0.479-1.071) | 0.104 |
|  | Grade 3 |  |  | 0.564 (0.386-0.825) | 0.003 | 0.565 (0.373-0.857) | 0.007 | 0.544 (0.350-0.847) | 0.007 |
| **Residence** (Urban=0) | Rural |  |  | 1.520 (0.746-3.095) | 0.249 | 1.492 (0.729-3.051) | 0.273 | 1.700 (0.799-3.619) | 0.168 |
| **Parental divorce** (No=0) | Yes |  |  | 0.868 (0.582-1.296) | 0.490 | 0.857 (0.574-1.279) | 0.449 | 0.860 (0.562-1.317) | 0.488 |
| **Father/mother deceased** (No=0) | Yes |  |  | 0.509 (0.061-4.260) | 0.533 | 0.508 (0.061-4.256) | 0.532 | 0.512 (0.057-4.639) | 0.552 |
| **Family structure**  (Two-parent family=0) | Single mother family |  |  | 1.489 (0.913-2.427) | 0.111 | 1.509 (0.924-2.462) | 0.100 | 1.437 (0.854-2.420) | 0.172 |
|  | Single father family |  |  | 2.395 (1.058-5.421) | **0.036** | 2.448 (1.076-5.570) | **0.033** | 1.886 (0.786-4.523) | 0.155 |
|  | Both parents absent |  |  | 2.444 (1.015-5.881) | 0.046 | 2.454 (1.016-5.929) | 0.046 | 2.484 (0.944-6.537) | 0.065 |
| **Only child** (No=0) | Yes |  |  | 0.859 (0.633-1.165) | 0.328 | 0.866 (0.637-1.177) | 0.358 | 0.962 (0.694-1.333) | 0.814 |
| **Father's education level**  (Primary school or below=0) | Junior high school |  |  | 2.507 (0.502-12.521) | 0.263 | 2.512 (0.504-12.531) | 0.261 | 3.154 (0.608-16.367) | 0.171 |
|  | High school or technical secondary school |  |  | 1.997 (0.396-10.079) | 0.402 | 2.014 (0.399-10.171) | 0.397 | 2.673 (0.504-14.173) | 0.248 |
|  | Bachelor or associate degree |  |  | 2.237 (0.440-11.370) | 0.332 | 2.258 (0.444-11.484) | 0.327 | 2.941 (0.552-15.670) | 0.206 |
|  | Master's degree or above |  |  | 2.341 (0.390-14.055) | 0.352 | 2.380 (0.396-14.308) | 0.343 | 3.616 (0.567-23.060) | 0.174 |
| **Mother's education level**  (Primary school or below=0) | Junior high school |  |  | 0.379 (0.084-1.701) | 0.205 | 0.392 (0.088-1.750) | 0.220 | 0.665 (0.138-3.207) | 0.611 |
|  | High school or technical secondary school |  |  | 0.426 (0.095-1.901) | 0.263 | 0.437 (0.098-1.938) | 0.276 | 0.796 (0.164-3.862) | 0.777 |
|  | Bachelor or associate degree |  |  | 0.326 (0.072-1.470) | 0.145 | 0.335 (0.075-1.507) | 0.154 | 0.555 (0.114-2.708) | 0.466 |
|  | Master's degree or above |  |  | 0.258 (0.045-1.488) | 0.130 | 0.266 (0.046-1.534) | 0.139 | 0.411 (0.064-2.629) | 0.348 |
| **Monthly household income per capita (yuan)** (<2000=0) | 2000-4000 |  |  | 0.691 (0.297-1.604) | 0.389 | 0.650 (0.278-1.519) | 0.320 | 0.744 (0.294-1.882) | 0.532 |
|  | 4000-6000 |  |  | 0.687 (0.289-1.630) | 0.394 | 0.653 (0.274-1.557) | 0.336 | 0.744 (0.288-1.921) | 0.542 |
|  | 6000-8000 |  |  | 1.073 (0.455-2.532) | 0.872 | 1.028 (0.433-2.438) | 0.950 | 1.230 (0.478-3.166) | 0.668 |
|  | 8000-10000 |  |  | 0.600 (0.247-1.460) | 0.260 | 0.570 (0.233-1.392) | 0.217 | 0.717 (0.271-1.897) | 0.503 |
|  | >10000 |  |  | 1.023 (0.437-2.395) | 0.957 | 0.982 (0.418-2.307) | 0.966 | 1.174 (0.460-3.000) | 0.737 |
| **Chronic disease** (No=0) | Yes |  |  |  |  | 0.473 (0.137-1.631) | 0.236 | 0.411 (0.112-1.503) | 0.179 |
| **Weight（kg）** |  |  |  |  |  | 1.001 (0.976-1.026) | 0.940 | 0.992 (0.966-1.018) | 0.544 |
| **Prefer sleeping with light on** (No=0) | Yes |  |  |  |  | 1.219 (0.803-1.851) | 0.352 | 1.219 (0.781-1.903) | 0.383 |
| **Eating after 9 PM** (Never=0) | Occasionally |  |  | 0.706 (0.311-1.601) | 0.404 | 1.043 (0.715-1.519) | 0.828 | 1.084 (0.728-1.614) | 0.692 |
|  | Every day |  |  | 0.739 (0.317-1.724) | 0.483 | 0.852 (0.346-2.101) | 0.728 | 1.063 (0.415-2.727) | 0.898 |
| **Baseline CDI-S** |  |  |  |  |  |  |  | 5.854 (4.215-8.131) | <0.001 |

*Note*: Model 1 is the unadjusted model;Note: Model 1 is the unadjusted model; Model 2 adjusts for demographic and family socio-economic factors; Model 3 further adjusts for health and behavioral factors based on Model 2; Model 4 further incorporates the corresponding baseline symptom levels based on Model 3.
