# Supplementary material for: Study on the association between sleep quality and anxiety–depression symptoms in school-aged children
Source: Front Public Health. 2026 Oct 20;14:1927475. doi: 10.3389/fpubh.2026.1927475 (PMC13727519; doi:10.3389/fpubh.2026.1927475)
Supplement: Supplementary file 15 [file Table_9.DOCX]

**Table S9. Longitudinal association between baseline sleep-related problems and follow-up coexisting anxiety and depressive symptoms (n=1405)**

| **Variable** | **Classification** | **Model 1** | | **Model 2** | | **Model 3** | | **Model 4** | |
| --- | --- | --- | --- | --- | --- | --- | --- | --- | --- |
|  |  | ***OR* (95% *CI*)** | ***P*** | ***OR* (95% *CI*)** | ***P*** | ***OR* (95% *CI*)** | ***P*** | ***OR* (95% *CI*)** | ***P*** |
| **Baseline PSQI-5** |  | 1.432 (1.332-1.540) | <0.001 | 1.428 (1.324-1.540) | <0.001 | 1.429 (1.331-1.541) | <0.001 | 1.147 (1.046-1.258) | 0.004 |
| **Sex** (Male=0) | Female |  |  | 0.918 (0.656-1.285) | 0.619 | 0.918 (0.684-1.293) | 0.625 | 0.879 (0.609-1.268) | 0.491 |
| **School** (School A=0) | School B |  |  | 0.978 (0.621-1.541) | 0.925 | 0.980 (0.663-1.543) | 0.931 | 0.911 (0.558-1.488) | 0.711 |
|  | School C |  |  | 0.677 (0.364-1.260) | 0.218 | 0.678 (0.441-1.263) | 0.221 | 0.644 (0.331-1.251) | 0.194 |
| **Grade** (Grade 1=0) | Grade 2 |  |  | 0.827 (0.534-1.280) | 0.394 | 0.822 (0.564-1.282) | 0.388 | 0.943 (0.590-1.507) | 0.806 |
|  | Grade 3 |  |  | 0.740 (0.477-1.146) | 0.177 | 0.732 (0.513-1.183) | 0.203 | 0.691 (0.411-1.161) | 0.163 |
| **Residence** (Urban=0) | Rural |  |  | 1.758 (0.817-3.779) | 0.149 | 1.735 (0.736-3.748) | 0.161 | 2.258 (1.008-5.057) | **0.048** |
| **Parental divorce** (No=0) | Yes |  |  | 0.784 (0.492-1.247) | 0.304 | 0.778 (0.504-1.239) | 0.291 | 0.739 (0.444-1.229) | 0.244 |
| **Father/mother deceased** (No=0) | Yes |  |  | 0.711 (0.082-6.162) | 0.757 | 0.722 (0.114-6.262) | 0.767 | 0.781 (0.079-7.751) | 0.833 |
| **Family structure**  (Two-parent family=0) | Single mother family |  |  | 1.577 (0.908-2.740) | 0.106 | 1.591 (0.875-2.766) | 0.100 | 1.268 (0.688-2.340) | 0.447 |
|  | Single father family |  |  | 2.302 (0.914-5.797) | 0.077 | 2.336 (0.787-5.902) | 0.073 | 1.805 (0.675-4.832) | 0.240 |
|  | Both parents absent |  |  | 2.060 (0.758-5.602) | 0.157 | 2.087 (0.693-5.697) | 0.151 | 1.709 (0.530-5.510) | 0.369 |
| **Only child** (No=0) | Yes |  |  | 0.755 (0.528-1.080) | 0.124 | 0.756 (0.548-1.083) | 0.127 | 0.727-(0.492-1.073) | 0.109 |
| **Father's education level**  (Primary school or below=0) | Junior high school |  |  | 3.089 (0.466-20.475) | 0.243 | 3.059 (0.446-20.110) | 0.245 | 2.983 (0.472-18.856) | 0.245 |
|  | High school or technical secondary school |  |  | 2.199 (0.328-14.762) | 0.417 | 2.189 (0.319-14.606) | 0.419 | 2.364 (0.363-15.395) | 0.368 |
|  | Bachelor or associate degree |  |  | 2.435 (0.359-16.509) | 0.362 | 2.421 (0.337-16.318) | 0.364 | 2.433 (0.370-15.994) | 0.355 |
|  | Master's degree or above |  |  | 3.094 (0.383-24.994) | 0.289 | 3.098 (0.344-24.893) | 0.288 | 3.874 (0.489-30.703) | 0.200 |
| **Mother's education level**  (Primary school or below=0) | Junior high school |  |  | 0.509 (0.097-2.666) | 0.424 | 0.523 (0.170-2.716) | 0.440 | 0.485 (0.088-2.676) | 0.407 |
|  | High school or technical secondary school |  |  | 0.626 (0.121-3.245) | 0.577 | 0.639 (0.214-3.282) | 0.592 | 0.744 (0.135-4.106) | 0.735 |
|  | Bachelor or associate degree |  |  | 0.452 (0.086-2.378) | 0.349 | 0.465 (0.154-2.428) | 0.364 | 0.474 (0.085-2.652) | 0.395 |
|  | Master's degree or above |  |  | 0.432 (0.063-2.981) | 0.394 | 0.443 (0.126-3.035) | 0.407 | 0.488 (0.066-3.612) | 0.482 |
| **Monthly household income per capita (yuan)** (<2000=0) | 2000-4000 |  |  | 0.665 (0.265-1.670) | 0.385 | 0.643 (0.308-1.629) | 0.352 | 0.694 (0.260-1.849) | 0.465 |
|  | 4000-6000 |  |  | 0.680 (0.263-1.760) | 0.427 | 0.662 (0.319-1.723) | 0.398 | 0.625 (0.227-1.724) | 0.364 |
|  | 6000-8000 |  |  | 0.982 (0.384-2.515) | 0.971 | 0.961 (0.457-2.479) | 0.934 | 1.024 (0.374-2.799) | 0.964 |
|  | 8000-10000 |  |  | 0.587 (0.220-1.567) | 0.288 | 0.568 (0.272-1.528) | 0.263 | 0.636 (0.223-1.812) | 0.397 |
|  | >10000 |  |  | 0.864 (0.339-2.202) | 0.079 | 0.846 (0.411-2.170) | 0.728 | 0.874 (0.323-2.363) | 0.791 |
| **Chronic disease** (No=0) | Yes |  |  |  |  | 0.708 (0.196-2.550) | 0.598 | 0.957 (0.264-3.467 | 0.947 |
| **Weight（kg）** |  |  |  |  |  | 1.002 (0.973-1.031) | 0.887 | 0.995 (0.965-1.026) | 0.749 |
| **Prefer sleeping with light on** (No=0) | Yes |  |  |  |  | 1.261 (0.762-2.028) | 0.338 | 1.414 (0.684-1.903) | 0.613 |
| **Eating after 9 PM** (Never=0) | Occasionally |  |  |  |  | 0.979 (0.619-1.504) | 0.922 | 0.969 (0.615-1.527) | 0.892 |
|  | Every day |  |  |  |  | 0.847 (0.331-2.367) | 0.752 | 0.878 (0.297-2.601) | 0.815 |
| **Baseline GAD-7** |  |  |  |  |  |  |  | 1.080 (1.028-1.134) | **0.002** |
| **Baseline CDI-S** |  |  |  |  |  |  |  | 1.242 (1.168-1.320) | <0.001 |

*Note*: Model 1 is the unadjusted model;Note: Model 1 is the unadjusted model; Model 2 adjusts for demographic and family socio-economic factors; Model 3 further adjusts for health and behavioral factors based on Model 2; Model 4 further incorporates the corresponding baseline symptom levels based on Model 3.
